# Toward Machine Learning Enhancement of Accelerated Surface Hopping with Scaled Spin–Orbit Couplings

**DOI:** 10.1021/acs.jpca.6c06078

**Published:** 2026-10-23

**Authors:** Jakub Martinka, Mahesh Kumar Sit, Pavlo O. Dral, Jiří Pittner

**Affiliations:** † Heyrovský Institute, 86875 Academy of Sciences of the Czech Republic, v.v.i., Dolejškova 3, 18223 Prague 8, Czech Republic; ‡ Department of Physical and Macromolecular Chemistry, Faculty of Science, Charles University, Hlavova 8, 128 43 Prague 2, Czech Republic; § State Key Laboratory of Physical Chemistry of Solid Surfaces, Department of Chemistry, College of Chemistry and Chemical Engineering, and Fujian Provincial Key Laboratory of Theoretical and Computational Chemistry, 12466 Xiamen University, Xiamen 361005, China; ∥ Institute of Physics, Faculty of Physics, Astronomy, and Informatics, Nicolaus Copernicus University in Toruń, ul. Grudziadzka 5, 87-100 Toruń, Poland; ⊥ Institute of Advanced Studies, Nicolaus Copernicus University in Toruń, ul. Wileńska 4, 87-100 Toruń, Poland; # Aitomistic, Shenzhen 518000, China

## Abstract

Surface hopping (SH) methods are typically employed to simulate ultrafast nonadiabatic processes, but long time scales often remain beyond their reach. To address this, the accelerated SH scheme mitigates this limitation by scaling the driving forces of such processes, either nonadiabatic couplings (NACs) in the case of internal conversion or spin–orbit couplings (SOCs) for intersystem crossing. However, obtaining the actual time constant requires extrapolation from several ensembles of trajectories with different scaling factors. This introduces a significant computational demand, often restricting the number of trajectories per ensemble and, therefore, reducing the statistical confidence in the resulting time constant. In this work, we investigate the accelerated scheme using silaethylene (CH_2_SiH_2_) as a case study, evaluating various population fitting methods and extrapolation techniques. We trained machine learning models for potential energy surfaces (PESs) and NACs, and extended our Rotate-Predict-Rotate approach to fit SOCs. These models demonstrate high performance, yielding populations within the confidence interval of the reference MR-CISD/SA-CASSCF­(2,2) data; however, the extrapolation itself is highly sensitive to the fitted time constants, leading to discrepancies in the final time constant. Finally, we showcase and discuss how ML models can enhance the reliability of an accelerated SH scheme.

## Graphical abstract

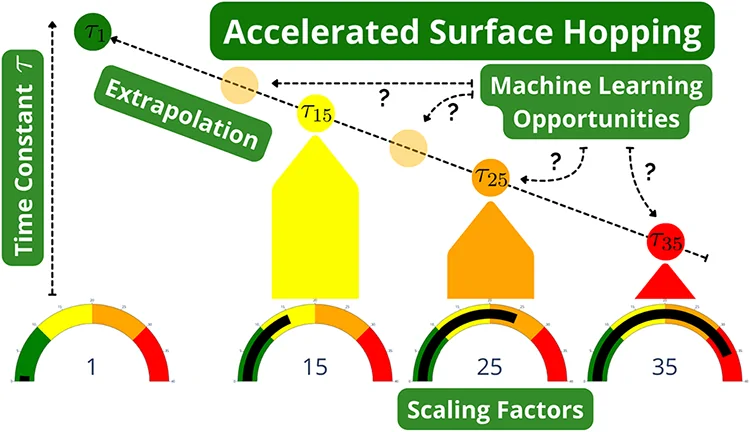

## Introduction

Photochemical and photophysical phenomena play a fundamental role in governing how molecules interact upon the absorption of light, enabling the conversion of absorbed energy into chemical and physical transformations. These processes are central to both natural systems and a wide range of technological applications, including photodynamic therapy,^1,2^ molecular solar thermal energy storage,^3^ and organic photovoltaics.^4,5^ Most tools probing these phenomena within the nonadiabatic molecular dynamics (NAMD) aim to describe short time scales only. This restriction is imposed by the need for high-accuracy excited-state potential energy surfaces (PESs), a demand that becomes even more significant when investigating larger chemical systems.^6^ While hardware improvements and algorithm optimizations continue to push these limits, long time scales pose additional challenges, e.g., stability of integration methods or accuracy of NAMD algorithms, and available methods remain in an early phase of development.^7^ However, if the objective of an NAMD study is to estimate a time constant or time scale, the couplings driving the process can be scaled to achieve acceleration;^8,9^ we refer to this as an accelerated scheme.

Within the variety of available NAMD methods, one of the most common is the trajectory surface hopping (SH), a member of the mixed quantum-classical family of algorithms that treats quantum and classical degrees of freedom separately.^10^ The most common variant of SH was introduced in the 1990s by Tully^11,12^ employing the fewest switches criterion, therefore abbreviated as FSSH. Originally derived to describe internal conversion (IC) processes only, the approach was later extended to account for transition between states of different spin multiplicity mediated by spin–orbit couplings (SOCs), i.e., intersystem crossing (ISC) processes.^13–21^ Nevertheless, the extension of FSSH to ISC presents several problems. For example, sum of hopping probabilities of all multiplets is not rotationally invariant, and when spin–orbit coupling is non-negligible, the PESs split, which must be accounted during dynamics propagation. Granucci et al. demonstrated that each spin multiplet cannot be treated as a single electronic state;^22^ unfortunately, even simpler schemes based on Landau–Zener type of SH does not perform well in these cases.^23^ One solution to these concerns involves propagating the dynamics in a spin-adiabatic (i.e., diagonal) representation obtained by diagonalizing of the total Hamiltonian matrix (see Methods section). However, this introduces numerical issues caused by arbitrary phases and unitary rotations among degenerate eigenvalues. To address this, it has been proposed to use the unitary matrix, which diagonalizes the total Hamiltonian matrix, to transform the state coefficients from diagonal basis to spin-diabatic (i.e., molecular Coulomb Hamiltonian (MCH)) representation, and several techniques have been introduced to achieve this effectively.^24–26^

In this work, we focus on silaethylene (CH_2_SiH_2_, see Figure 1a), which has been studied previously both statically and dynamically, but never with inclusion of ISC relaxation to the triplet state. At the optimized geometry, the ππ* is the lowest singlet state; the Rydberg state π-3s is located only 0.6 eV above, but destabilized, and other states are excluded as they lie significantly higher in energy.^27^ Thus, only the first two singlet states are included in the SH dynamics, following ref 28. Silaethylene was studied at MR-CISD level-of-theory including two lowest singlet states within the FSSH scheme, where two major relaxation pathways, Type T and Type B, were identified.

**Figure 1:**
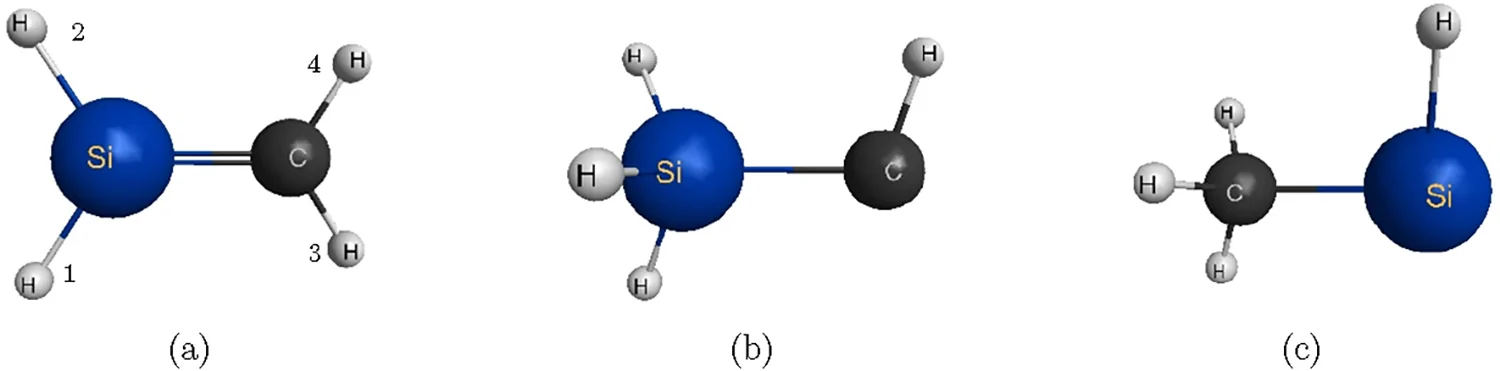
Optimized geometries of (a) CH_2_SiH_2_, (b) SiH_3_CH and (c) CH_3_SiH.

We have recently compared the MR-CISD energies of silaethylene against Mukherjee’s multireference coupled cluster theory incorporating triples perturbatively (MR-CCSD­(T)), as well as the multireference averaged quadratic coupled-cluster (MR-AQCC) level-of-theory.^29^ Geometry optimization revealed a triplet ground state of SiH_3_CH diradical (Figure 1b) and a singlet ground state of CH_3_SiH diradical (Figure 1c), the latter of which is energetically close to optimized *S*_0_ state. Furthermore, structures related to rotation of the CH_2_–SiH_2_ bond are reported to be energetically degenerate. Silaethylene is a small molecule, which allows for the use of a high level-of-theory for its description, while the small magnitude of SOCs (see Figure S9) indicates a slow transfer to the triplet state. The estimation of time constant is, therefore, beyond the scope of standard FSSH including ISC, making it a perfect candidate for testing an accelerated FSSH scheme; specifically, by scaling the SOCs that drive the process.

In recent years, artificial intelligence (AI) has found a breathtaking applications in chemistry, ranging from protein structure predictions,^30^ self-driving laboratories,^31^ chemical large language models,^32^ up to AI agents autonomously performing computational chemistry simulations.^33,34^ Apart from these exciting new developments, machine learning (ML) methods have been part of computational chemistry for many years, finding applications particularly in ground-state molecular dynamics (MD) simulations.^35–40^ Their advantage lies in the fact that, once trained on a certain level of electronic structure data, the same accuracy is in principle achieved during propagation, but with a significant acceleration. Furthermore, physical constrains can be encoded into the design of ML model, increasing both performance and robustness.

Despite the great success of ML models for ground-state MD, describing excited-state events is far from simple, as excited PESs pose challenges even for standard electronic structure methods. Electronic states become degenerate at conical intersections (CIs), where PESs become nondifferentiable; a significant problem for ML methods that are designed to fit smooth functions. Nevertheless, the use of ML methods within NAMD has become a research focus for several groups.^41–50^

In this work, we combine our previous efforts on learning vectorial properties and phase-correction workflows of learning nonadiabatic couplings (NACs), applying them to fit SOCs using silaethylene as a test case. This work demonstrates the generality of the Rotate-Predict-Rotate (RPR) scheme as well as the learning procedure of targets with arbitrary phases. This approach significantly accelerates NAMD simulations involving slow ISC to the triplet manifold. We conduct an analysis of accelerated FSSH scheme and critically discuss the opportunities of ML application within an accelerated SH schemes.

## Methods

### Trajectory Surface Hopping Involving Spin–Orbit Effects

To account for ISC, one must include spin–orbit effects in the Hamiltonian. The total Hamiltonian *Ĥ* ^tot^ is expressed as the sum of the molecular Coulomb Hamiltonian

$${Ĥ}^{\mathrm{MCH}}=-\sum_{i}\frac{\hbar^{2}}{2m}\nabla_{i}^{2}+\frac{e^{2}}{4\pi \epsilon_{0}}\left(\sum_{A<B}\frac{Z_{A}Z_{B}}{R_{\mathrm{AB}}}-\sum_{i}\sum_{A}\frac{Z_{A}}{R_{\mathrm{iA}}}+\sum_{i<j}\frac{1}{r_{\mathrm{ij}}}\right)$$ 1

and the Breit-Pauli spin–orbit Hamiltonian,

2 $${Ĥ}^{\mathrm{SO}}=\frac{1}{8\pi {\text{ϵ}}_{0}m^{2}c^{2}}\left(\sum_{i}\sum_{A}\frac{Z_{A}e^{2}}{r_{\mathrm{iA}}^{3}}{\mathrm{l̂}}_{\mathrm{iA}}\cdot {ŝ}_{i}-\sum_{\mathrm{i,j}\neq i}\frac{e^{2}}{r_{\mathrm{ij}}^{3}}{\mathrm{l̂}}_{\mathrm{ij}}\cdot \left({ŝ}_{i}+2{ŝ}_{j}\right)\right)$$

The matrix elements of this Hamiltonian between components of spin multiplet states can be expressed using the Wigner-Eckart theorem in terms of reduced matrix elements which form an axial Cartesian SOC vector.^51^ We apply ML to the SOC vector, using the RPR procedure to maintain its covariance (improper rotations are not included in RPR, so the axial character does not matter). In particular, the singlet–triplet SOC matrix elements are identical to the components of the SOC vector, but this approach can be generalized to treat arbitrary spin multiplicity.

In the Tully’s FSSH scheme, the coefficients in MCH basis {ϕ_*i*_} are propagated according to

3 $$\frac{dc_{k}\left(t\right)}{\mathrm{dt}}=\sum_{j}\left(-iH_{\mathrm{kj}}^{\mathrm{tot}}-\sigma_{\mathrm{kj}}\right)c_{j}\left(t\right)$$

where σ_*kj*_ = ***R* ˙**(*t*)·⟨ϕ_*k*_|∇_***R***_|ϕ_*j*_⟩ is the time-derivative coupling, expressed as the scalar product of the velocity and the NAC, and *H*_*kj*_^tot^ is the matrix element of the total Hamiltonian, which is typically diagonal. However, Granucci et al. demonstrated that the transition probabilities in the MCH representation are not rotationally invariant.^22^ Furthermore, when SOCs are non-negligible, the dynamics should be performed in the basis of the eigenvectors {ψ_*i*_} of the total Hamiltonian, i.e., diagonal representation. This involves using a unitary matrix ***U*** to diagonalize the total Hamiltonian matrix and to transform the quantities in eq 3. Unfortunately, this is not trivial due to the occurrence of d ***U***/d*t* in the equations of motion, which causes numerical instabilities. Solutions to this problem have been proposed, such as the 3-step propagator approach by González et al.,^25^ or the removal of redundant degrees of freedom from ***U*** before taking the derivative by some of us.^26^ In this work, we employ the former approach, which transforms the coefficients in the diagonal basis *c̅*_*i*_ to the MCH basis as

4 $$c_{i}=\sum_{j}U_{\mathrm{ji}}{\mathrm{c̅}}_{j}$$

followed by propagation in the MCH basis analogously to eq 3, and finally back-transformation to the diagonal representation. A hop from state *k* to *j* is performed according to probability *p* given by

5 p(k→j)=(1−|c̅k(t+Δt)|2|c̅k(t)|2)×Re(c̅j(t+Δt)Pjk*c̅k*(t))|c̅k(t)|2−Re(c̅k(t+Δt)Pkk*c̅k*(t))

where *P*_*ij*_ is an element of the propagator matrix defined as

6 $$P\left(t+\Delta t,t\right)=U^{†}\left(t+\Delta t\right)\exp\left(-\Delta t\left(iH^{\mathrm{tot}}-\sigma \right)\right)U\left(t\right)$$

A hop is initiated when a uniformly generated random number *r* ∈ [0,1] satisfies the condition

7 $$\sum_{i=1}^{j-1}p\left(k\to i\right)<r\leq \sum_{i=1}^{j}p\left(k\to i\right)$$

### Accelerated Surface Hopping Scheme

The most computationally demanding step in SH is solving the electronic Schödinger equation, which inherently limits NAMD to simulating phenomena at hundreds of femtoseconds time scales, depending on the size of the system, basis set, and level of theory. While long time scales have been probed for different NAMD schemes in recent years,^7,8,43,44,52–54^ trajectory-based methods can suffer zero-point energy leakage when using Wigner-based initial conditions.^55^ Fortunately, if the goal of an NAMD study is to determine the time constant or lifetime, it is possible to use an accelerated SH scheme. This is based on artificially scaling the couplings that drive the process of interest by a constant α > 1, i.e., ISC driven by SOCs as

8 $${Ĥ}_{\mathrm{ij}}^{\alpha }=\alpha \left\langle \phi_{i}\right|{Ĥ}_{\mathrm{SO}}\left|\phi_{j}\right\rangle$$

The selected α values should be small enough not to violate the short-time approximation; however, very small α values may lead to difficulties in fitting the population dynamics. The appropriate choice is system-specific, as it is influenced by the magnitude of the couplings and the energetic proximity of the states. In practice, they are typically selected empirically by running several trajectories to determine whether ISC takes place.

Theoretical treatments of the ISC processes usually assume a two-state model with an initial state ⟨ϕ_*i*_| and a final state |ϕ_*f*_⟩, while the weak coupling limit ⟨ϕ_*i*_|*Ĥ*_SO_|ϕ_*f*_⟩≪ |*E*_*i*_^el^ – *E*_*f*_^el^| guarantees that the SOC is smaller than the electronic energy gap, and that no other side channels exist. Obviously, the two-state assumption can only be fulfilled approximately, when other states are energetically well separated. Under these assumptions, first order perturbation theory applies and the Fermi golden rule can be used for the transition rate^56^

9 $$k_{\mathrm{ISC}}=\frac{2\pi }{\hbar }\left|\left\langle \phi_{i}\right|{Ĥ}_{\mathrm{SO}}\right|\phi_{f}\rangle {|}^{2}\delta (E_{i}-E_{f}).$$

When scaling the couplings ⟨ϕ_*i*_|*Ĥ*_SO_|ϕ_*f*_⟩ by a factor α, the time constant τ, which is inversely proportional to *k*_ISC_, thus scales with α^–2^,

10 $$\tau^{\alpha }=\frac{A}{\alpha^{2}}$$

which we refer to as the Fit1 method throughout the text. However, eq 10 holds only for a two-state system; the general *N*-state relationship is not known; when scaling NAD coupling in ref 57 the authors assumed a rational fraction form with additional constant parameter in the denominator. We did not employ this fitting formula as it would lead to nonzero ISC rate for SOC scaled to zero.

The time constant or lifetime is obtained by extrapolation to α = 1, which requires running multiple ensembles of trajectories (possibly with different initial conditions) for a set of scaling constants to determine *A*. In a typical accelerated SH study, we are unfortunately limited to using only a few different scaling factors. Equation 10 correctly describes the limit of scaling SOC to zero, leading to the divergence of τ^α^, i.e., zero ISC rate. However, the asymptotic behavior of eq 10 for large scaling parameter α leading to infinitely fast ISC (zero τ^α^) is unphysical, since at some point the scaled SOC will become so big that one leaves the weak coupling limit and other factors will become the rate limiting bottlenecs. To alleviate this problem, eq 10 can be extended by introducing an additional parameter, τ_∞_, so that the model reproduces the expected limiting behavior, with the time constant diverging as α → 0 and approaching the finite value τ_∞_ as α → ∞,

11 $$\tau^{\alpha }=\frac{A}{\alpha^{2}}+\tau_{\infty }$$

Another approach is to fit the data on a log–log scale using eq 10, i.e., using a linear function *C*log­(α) +*D*, where *D* corresponds to log*A* and the slope *C* is optimized but expected to be near −2, consistent with eq 10. This approach is referred to as the Fit2 method and, unlike Fit1, does not restrict the exponent of α to be −2, although, as is the case of τ_∞_, the additional fitting parameter generally increases the uncertainties and widens the confidence intervals.

### Machine Learning Driven Trajectory Surface Hopping

In supervised learning, an ML model maps input features, typically represented by a molecular descriptor, to targets, i.e., molecular property of interest. There is a variety of molecular descriptors, commonly divided into global types, e.g., relative-to-equilibrium (RE),^58^ Coulomb matrix (CM)^59,60^ and others, or local, e.g., smooth overlap of atomic positions (SOAP)^61^ and others. Furthermore, one can combine a descriptor derived from molecular geometry with an additional property if it is available and correlates with the target.

The choice of ML regressor is another nontrivial task, as models are generally categorized into groups of neural networks and kernel methods, each containing different architectures. We limit ourselves to well-established approaches within the MLatom package,^62^ specifically the multistate ANI^63^ (MS-ANI) model and kernel ridge regression (KRR) with Gaussian kernel. We point the reader to the relevant reviews discussing the strengths and weaknesses of various ML approaches (e.g., refs 64–68 .).

Data set construction is a crucial part of any successful ML application. An active learning (AL) procedure, based on uncertainty quantification, iterative data augmentation and model retraining, might be an ideal solution, but currently we do not have deep integration with the NAMD code with ISC adaptation, which is one of our future goals. In addition, AL still requires manual experimentation such as careful selection of system-specific thresholds, although progress has been made in this direction.^43,63^ Once reference trajectories are available, a training set may be created by random selection, assuming that all the relevant PES regions and geometries are well represented. Rather than building the training set from full trajectories, selecting snapshots from across the entire data pool may be preferred.^69^

ML-driven FSSH accounting for IC and ISC relaxation requires the PESs and their gradients, as well as NACs and SOCs. While fitting PESs is a well-established task for which robust AL schemes exists,^43,63,70,71^ NACs and SOCs possess different challenges. NACs are vectorial, diverge at CI, and can have an arbitrary sign due to the wave function phase. Conversely, SOCs are typically smooth, delocalized and can be complex-valued; but are also vectorial and can have arbitrary sign. Learning vectorial targets is problematic because ML models must satisfy rotational equivariance. Several approaches have been proposed to address this, such as explicitly building equivariance into the model architecture^72–74^ or using RPR approach developed by some of the authors of this study.^75^ The latter has proved to be a simple and efficient technique to fit dipole moments and polarizabilities, and NACs in the context of NAMD.^76^ It relies on training the model on a prealigned data set; during the inference stage, the molecular structure is rotated to a reference frame before the ML prediction, and the resulting vectorial property is then back-rotated to the original orientation. Training is further complicated by arbitrary sign in the training database; these can be preprocessed before training,^43,76–78^ or alternativelty, a phase-less loss can be employed in the case of neural networks.^79^ Additionaly, commonly used descriptors were designed for learning PESs and might perform poorly for other properties.^76^

## Results and Discussion

### Reliability of the Accelerated Surface Hopping Scheme

Before discussing opportunities for ML applications, we first examine the accelerated SH scheme based on quantum chemical method for silaethylene, involving two singlet states (*S*_0_, *S*_1_) and one triplet (*T*_1_). We use the same settings for static calculations as in ref 29., specifically MR-CISD/SA-CASSCF­(2,2) with aug-cc-pVDZ basis set in COLUMBUS (version 7.2, 2022),^80,81^ which we abbreviate to MR-CISD further in the text for simplicity. The 500 initial conditions for the dynamics were generated using the Wigner distribution of a quantum harmonic oscillator^82^ for each normal mode from the optimized ground-state geometry at 298 K. From these, 200 trajectories were propagated for each of the scaling factors 15, 25 and 35, i.e., 600 trajectories in total. Once the ML models were trained, the ensembles were extended to cover all 500 initial conditions to generate 1,500 trajectories in total. In the case of ML trajectories, we also tested independent sets of initial conditions (500 trajectories per α value; see Figure S14) to mitigate potential bias, as well as different numbers of trajectories for each scaling factor (α = 15:700, α = 25:600, α = 35:500; see Figure S15) to increase the confidence in the results obtained for smaller α values. These tests may be relevant for assessing the robustness of the accelerated SH scheme; however, we do not find any significant discrepancies that would affect the conclusions reported for the same set of initial conditions.

We employed our ISC adaptation of Newton-X,^26,83,84^ with all trajectories initiated in *S*_1_. The final time of the dynamics was set to 500 fs, with a nuclear time step of 0.5 fs and an integration step for the electronic Schrödinger equation of 0.025 fs. The dynamics were performed using 3-step propagator approach^25^ in the diagonal representation. The electronic state coefficients were transformed to the MCH representation to obtain corresponding populations. Following a successful hop, the momenta were rescaled along the NACs to ensure conservation of the total energy. In case of a frustrated hop, the momenta were left unchanged. No decoherence correction was applied because existing decoherence schemes have primarily been developed for systems with rapidly decaying NACs, whereas their applicability to dynamics involving significant SOCs remains uncertain.^22,85,86^ In the plots showing populations, the average of |*c*_*i*_|^2^ is plotted; its validity was confirmed by comparing it against the singlet and triplet composition of the active PES, averaged over all trajectories, as well as against dynamics with phase correction (see Figure S25). Out of 200 MR-CISD trajectories, an average of 12 terminated shortly before the end of the propagation due to inconsistent energies or transition densities reported by Columbus. The ML-driven trajectories were free of such failures.

While accelerated NAMD schemes have been applied to both NACs^57,87^ and SOCs.^9,88,89^ The primary objective of this work is to study the ISC process; we therefore apply the scaling factor exclusively to SOCs. The SOCs facilitate the transition to the triplet state, represented by the increase in its average adiabatic population (see Figure 4a). This population is summed over all triplet components and fitted using *A* ^pop^(1 – exp (−*t*/τ^α^)), where the *A* ^pop^ constant corresponds to the asymptotic maximum triplet population. This choice is rationalized by the plateau in *T*_1_ population for sufficiently large α (see Figure S8). However, more complex population transfer can be observed in the general case. In such cases, an advanced fitting procedure based on an appropriate kinetic model should be employed to obtain reliable scaled and extrapolated time constants.

Rather than fitting each ensemble of trajectories independently, we employ a multicurve fitting strategy that enforces the constant *A* ^pop^ to be identical across data corresponding to different scaling factors α. The multicurve fitting improves the numerical stability of the fits and robustness of the overall extrapolation part of the accelerated scheme (see Figure S7 without multicurve strategy and the corresponding section of the Supporting Infomation (SI)). For each population fit, we perform 5000-sample bootstrapping^90–92^ by randomly selecting *n* trajectories with replacement to evaluate the uncertainty associated with the fitted parameters. The resulting values are then used to define confidence intervals for the extrapolated time constants.

The use of an accelerated SH scheme with scaling of couplings inherently requires several ensembles of trajectories to obtain confident population fits for each scaling factor. These fits provide time constants that can be used for extrapolation to a scaling factor of 1 using the Fit1 and Fit2 methods. We also compare the approach using τ_∞_ in the case of 500 ML trajectories (see Figure S12), as well as for the 200 MR-CISD trajectories (see Figure S13). For *N* ensembles, *N* times more trajectories must be propagated, which significantly increases the computational demand; consequently, the number of trajectories *n* within each ensemble is typically sacrificed in standard accelerated SH studies. Using three ensembles of MR-CISD trajectories, we analyzed the uncertainty introduced by the number of trajectories *n* in each ensemble. Figure 2 illustrates the extrapolation to scaling factor of 1 based on different values of *n*, ranging from 50 to 200 trajectories. The extrapolated time constants using the Fit1 (Figure 2a) and Fit2 (Figure 2b) methods are compared. The top-right plot shows the extrapolated time constants obtained from an analysis of the first *n* trajectories; specific values (in ps) are displayed in the bottom-left. In the Fit1 method, where the exponent is fixed to the theoretical value of −2, the extrapolated time constants decreases and slowly converges to 434 ps for an ensemble of 200 trajectories. The Fit2 method, which fits points in log–log scale and does not restrict exponent, converges to 225 ps for 200 trajectories. The slope ranges from −1.5 to −2.1 (the blue curve on top right of Figure 2b), but once at least 110 trajectories are included in the analysis it fluctuates around slope value of −1.84. Regardless of the fitting method, our analysis suggests that at least 100 trajectories per ensemble should be included to obtain reliable estimate of the time constant at α = 1. Same conclusion is obtained while analyzing trajectories based on ML models (see Figure S3).

**Figure 2:**
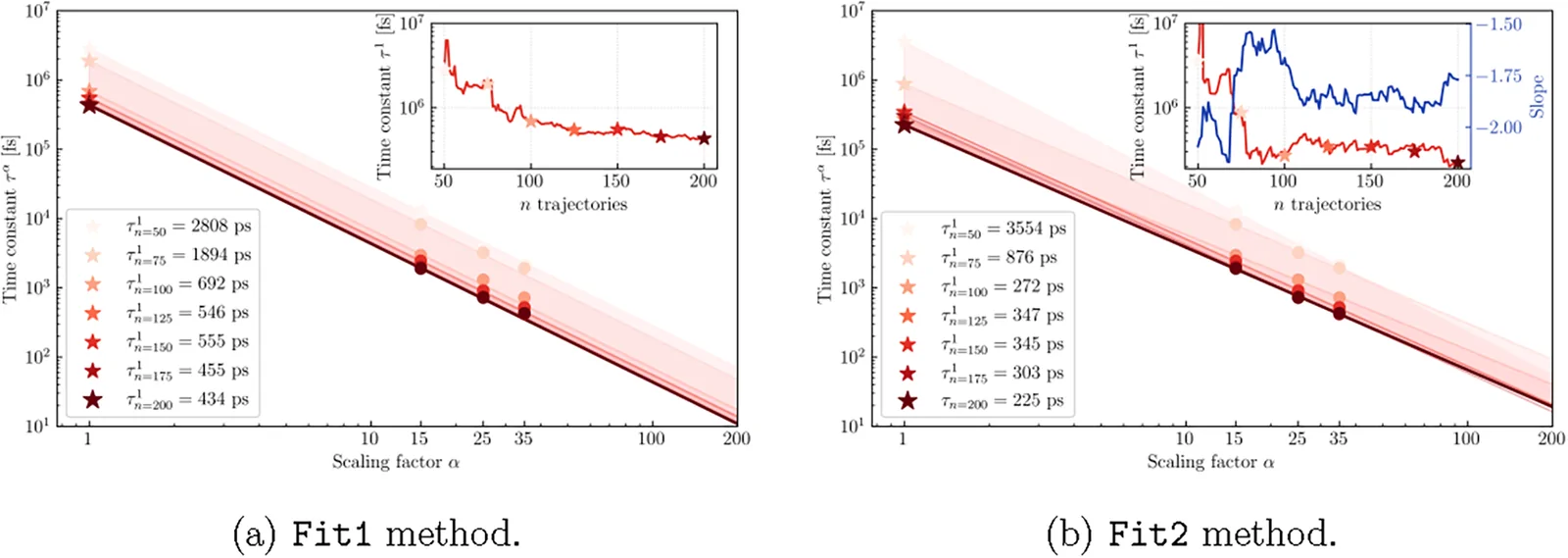
Extrapolation of MR-CISD time constants to α = 1 using Fit1 and Fit2 methods, based on the number of trajectories *n*. The top-right plot shows the time constants obtained by analyzing *n* trajectories; in the case of Fit2, the slope is shown in blue. Scaling factors of 15, 25, and 35 were used.

The requirement for multiple ensembles, each comprising hundreds of trajectories, presents an opportunity for ML to increase confidence of SH schemes accelerated by scaled couplings. We first briefly discuss approaches that turned out problematic and were thus not followed and analyzed in further detail. One natural idea for ML application is to increase *N* by generating more ensembles with different scaling factors α, thereby providing more points for extrapolation to 1. However, this proved difficult for smaller values of α due to the numerical challenge of fitting shallow, slowly increasing triplet populations, which in general results in large bootstrap errors, as seen for the population fits. Furthermore, extending such trajectories beyond the reference time scale (500 fs in our case) may cause instabilities in the dynamics due to extrapolation outside the training set; this is strongly system- and process-specific and might eventually be resolved by advanced sampling technique. On the other hand, using ensembles with larger α is not recommended, as it may negatively influence the multicurve fit and the extrapolated time constants. Finally, increasing *N* within the existing range of scaling factors, e.g., using {15, 20, 25, 35} instead of {15, 25, 35}, does not significantly affect the resulting time constant when compared to the uncertainty related to a small *n* (see Figure S1). We therefore conclude that the most promising use of ML in an accelerated SH scheme is to expand the size of the trajectory ensembles themselves.

### Evaluating Accuracy of Machine Learning Models

When applying ML models in production simulations, their performance is typically evaluated on an independent, hold-out test set, unseen during model training or selection. To construct this data set, we used 100 MR-CISD trajectories with scaling factor 20, generating a randomly selected pool of 6000 points, of which 5000 were used for training and 1000 for testing. We note that this approach might not be ideal for other systems in general, as the use of scaling factors may drive the system toward different regions of configurational space. To reduce the risk of ML models entering the extrapolation regime, the training and test sets should be assembled from trajectories with different scaling factors, ideally within an active learning framework. For silaethylene, considering two singlet states and one triplet state, the models must describe three adiabatic energies, corresponding gradients, one NAC between singlet states and two SOCs.

The most critical aspect is the accurate learning of PESs, as they govern the dynamics and thus determine the exploration of the configurational space. We trained an MS-ANI model for the *S*_0_ and *S*_1_ PESs, while the *T*_1_ surface was fitted using a separate ANI model on the same training set. All models achieved satisfactory performance on the test set, with *R* ^2^ values exceeding 0.95 for *S*_1_, 0.98 for *S*_0_, and 0.99 for *T*_1_. Therefore, a crucial part of the evaluation involves focusing on the NAMD simulations themselves and their trajectory analysis.

In case of silaethylene, the conical intersection is reached through the torsion around C–Si bond.^27^ Furthermore, the C–Si stretch and symmetric bipyradimidalization were previously reported as the main modes playing role in the *S*_1_ deactivation.^28^ Once the symmetric bipyramidalization is the main motion driving the relaxation, the trajectory is called type B. When the torsion around the C–Si dominates, the trajectory is regarded as type T. These two modes can be characterized by additional coordinates, θ and β, defined as

12a $$\theta =\sqrt{\frac{\theta_{1}^{2}+\theta_{2}^{2}}{2}}$$

12b $$\beta =\sqrt{\frac{\beta_{1}^{2}+\beta_{2}^{2}}{2}},$$

where θ_1_ (H_1_–Si–C-H_3_) and θ_2_ (H_2_–Si–C-H_4_) dihedral angles capture the central torsion, whereas, β_1_ (C–H_1_-H_2_–Si) and β_2_ (Si–H_3_-H_4_–C) describe the relative orientation of the methylene and silylene groups (see Figure 1a).

The projection of MR-CISD and ML trajectories onto the β – θ plane provides an ideal test for the ML-PESs. These two modes exert a strong influence on the dynamics during the early stage, and the trajectories can be shown to be separated by the β = 0.95·θ line (see Figure 3). Type T trajectories are characterized by simultaneous torsional and Si–C stretching. The latter lifts the degeneracy, causing the trajectories to hop later compared to the type B trajectories, which are driven by the Si–C stretch into the region of the bipyramidalized crossing seam.^28^ Type B trajectories reach the crossing seam faster than type T, meaning that the ratio between the two modes must be reproduced by the ML models to achieve agreement with the reference *S*_1_ population decay. As evident from Figure 3, the MR-CISD and ML trajectories agree well when projected onto the β – θ plane up to 20 fs. In case of the 200 MR-CISD trajectories, 72% trajectories are classified as type T and 28% as type B. In comparison, the ML trajectories contain 65% type T and 35% of type B. This difference is caused by trajectories that land in proximity to the β = 0.95·θ line within 20 fs, which alters the classification ratio. However, both distributions are close to the previously reported ratio of 68 and 32%.^28^ This indicates that the *S*_1_ → *S*_0_ deactivation channel is well recovered, which will be demonstrated on *S*_1_ decay in Figure 4a.

**Figure 3:**
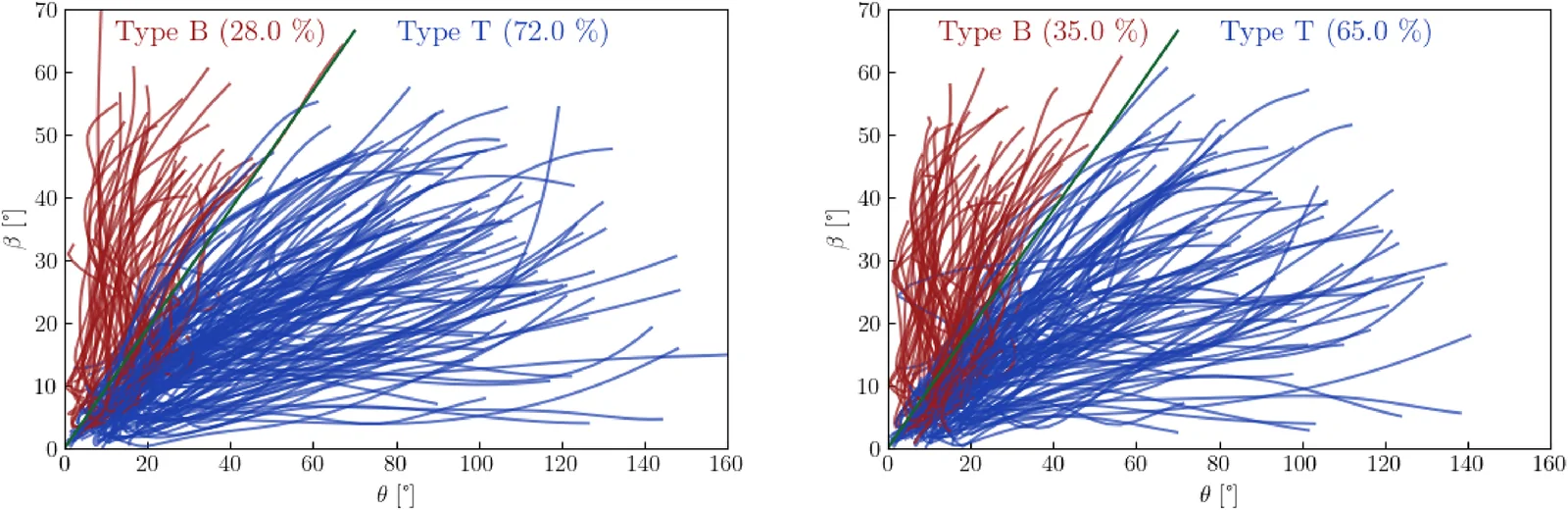
Projection of MR-CISD (left) and ML (right) trajectories with scaling factor α = 15 onto β – θ plane up to 20 fs. The deactivation process of silaethylene is well recovered by ML-driven trajectories. Scaling factor of SOCs does not influence this projection in both cases (see Figures S4 and S5).

**Figure 4:**
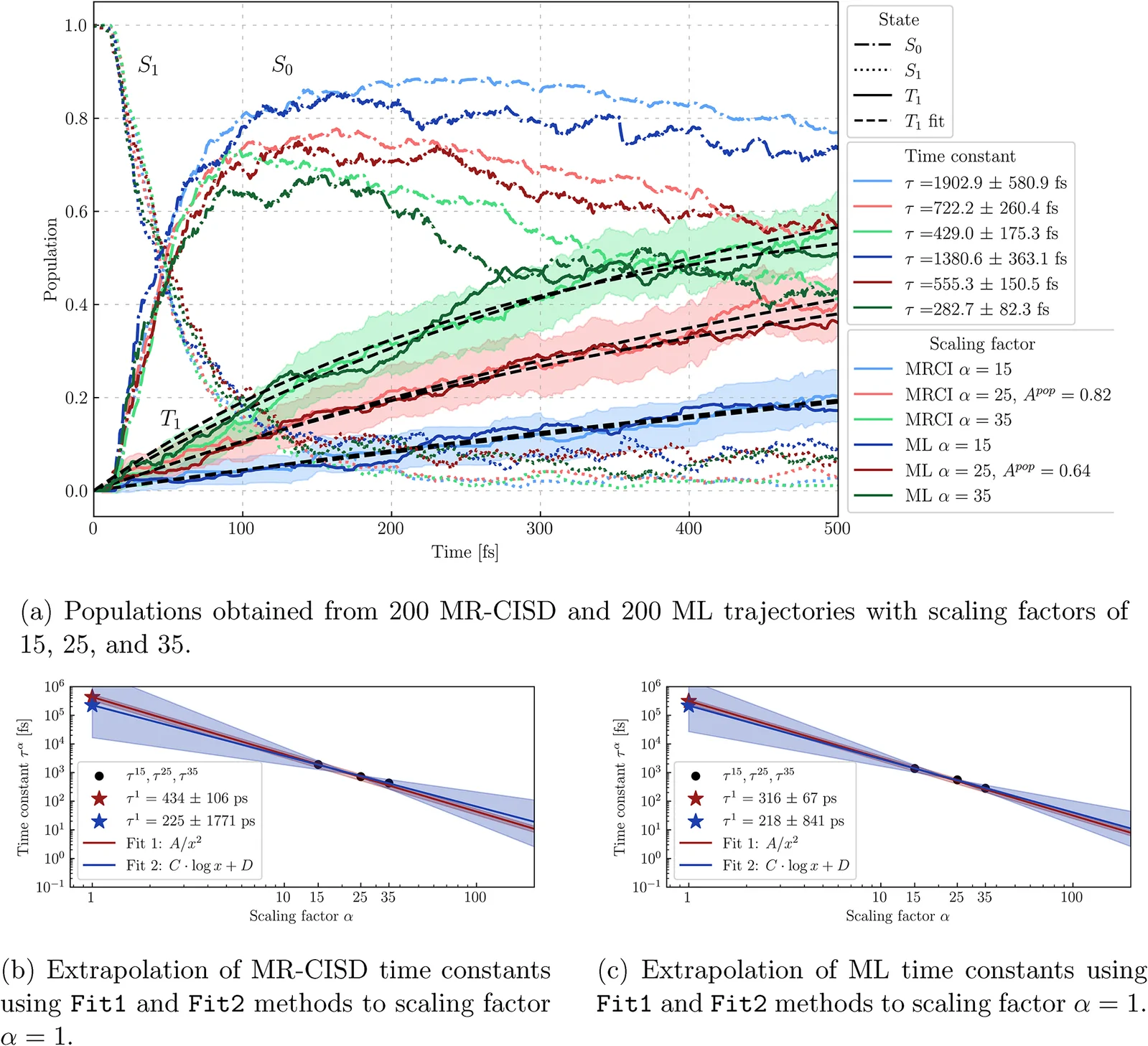
Comparison of MR-CISD and ML results from FSSH simulations of silaethylene. All trajectories were initiated in *S*_1_, and the deactivation to *S*_0_ is well reproduced. The transition to *T*_1_ falls within the confidence interval (a). However, the *T*_1_ fits are highly sensitive, leading to discrepancies when extrapolated to a scaling factor of 1 (b, c).

The NACs between *S*_0_ and *S*_1_ were fitted using KRR with RPR approach and the gradient difference as a descriptor. Prior to fitting, we followed the established protocol of multiplying the NACs by the energy gap Δ*E*_01_ to remove nonsmooth behavior^60^ and applied phase-correction to the data set using iterative procedure described in ref 76. SH simulations with the NAC model are expected to be sensitive to the minute changes in the model quality and, hence, we adopt a special model selection strategy based on the lessons for similar cases from the a previous work by one of us.^93^ The key lesson is that among many models with small errors in the hold-out test set, some perform very well in simulations and some very badly. Hence, test errors are insufficient and a proxy, cheap-to-compute property, which is connected to what is important in the final simulations, can be selected to evaluate the model quality before running the simulations. In our case, such proxy-property can be the error in NACs, in the nonadiabatic regions (where NACs are nonzero), i.e., for small Δ*E*_01_. To address this while maintaining simplicity, we constructed a dedicated “selection set” with 1000 points from the 5000-point pool by taking only the geometries with smallest Δ*E*_01_, yielding a diverse set of configurations with a relatively small energy gap, which is complementary to and not overlapping with the hold-out test set. The training set was generated by repeatedly sampling 5000 points using different random seeds, followed by training separate ML models on each subset. The models were evaluated on the 1000-point selection set, and the best-performing one was retained for subsequent dynamics simulations.

We verified that *R* ^2^ exceeding 0.9 was achieved on the hold-out test set. However, as mentioned above, such static metrics are not decisive and do not necessarily reflect the performance in the production simulations.^93^ A more reliable assessment requires evaluating the model within dynamical simulations, where preliminary results indicate satisfactory performance.

The SOCs were trained following a procedure similar to that used for the NACs, utilizing RPR with phase-correction. However, in contrast to NACs, SOCs are smooth and typically slowly varying, which eliminates the need for multiplication by the energy gap and requires the use of a different descriptor. We benchmarked several rotationally invariant descriptors, such as RE, CM, SOAP; however, the best performance was unexpectedly obtained using Cartesian coordinates translated to the center of mass and rotated into a standard orientation via RPR based on tensor of inertia (for detailes see ref 75.). This somewhat counterintuitive result is consistent with our previous experience, where an RE descriptor augmented with selected Cartesian coordinates performed best for dipole moment prediction. As with the NAC model, such comparisons should be interpreted with caution, as the ultimate validation lies in reproducing reference dynamics. While the model predicting SOCs between *S*_0_ and *T*_1_ achieved good performance (*R* ^2^ over 0.98) on the randomly selected test set, comparable accuracy could not be obtained for the *S*_1_ – *T*_1_ SOCs. Nevertheless, the *S*_1_ – *T*_1_ SOCs are extremely small, and setting them to zero along the entire trajectory resulted in an indistinguishable population dynamics. This indicates that their contribution is negligible and that the reduced accuracy of the corresponding model does not affect the overall dynamical outcomes.

### Machine Learning Models Reproducing Reference Trajectories

The final test of the ML models is their performance within SH simulations facilitated by the ISC-adapted Newton-X interfaced with MLatom, using the same simulation parameters and initial conditions as the MR-CISD reference. The resulting ML populations are plotted in Figure 4a (dark lines), alongside the MR-CISD populations (light lines) discussed previously. By investigating the state coefficients in the MCH basis from the 200 MR-CISD trajectories for all α values (15, 25 and 35) we collected configurations related to changes of the *T*_1_ state within an interval of 0.5 to 0.95. It can be seen from individual trajectories that ISC is mainly described as *S*_0_ → *T*_1_, while majority of selected configurations are associated with a CH_2_–SiH_2_ angle of 90 deg (see Figures S16–S18), small *S*_0_ – *T*_1_ energy difference (see Figures S19–S21), and relatively large SOCs (see Figures S22–S24). Furthermore, the slow ISC is explained by an energy barrier associated with accessing configurations with large SOC (see Figures S12 and S13).

The overall population transfer is well-described by the ML models, given the relatively simple procedure used to assemble the training sets. The *S*_1_ decay is in excellent agreement with the MR-CISD reference, indicating that the ML-PESs and ML-NACs achieved satisfactory performance. However, it is apparent that the ML populations are not as smooth as the reference; this could be improved by increasing the number of trajectories *n* within an ensemble; the primary motivation for using ML models, which are significantly faster than MR-CISD for trajectory propagation. Furthermore, after 150 fs the *S*_1_ state remains more populated in the ML results, suggesting potential for further refinement. This could potentially be resolved through an AL; however, such an approach requires a complex integration of ISC-adapted NAMD and ML codes, which is beyond of scope of this work.

Despite the ML *T*_1_ populations falling within the reference MR-CISD error margin ϵ­(*t*) of the 95% confidence interval calculated as

13 $$\epsilon \left(t\right)=1.96\sqrt{\frac{p_{T_{1}}\left(t\right)\left(1-p_{T_{1}}\left(t\right)\right)}{N_{\mathrm{traj}}}}$$

the multicurve fits result in MR-CISD time constants of a 1903 ± 581, 722 ± 260 and 429 ± 175 fs, compared to 1381 ± 363, 555 ± 151, 283 ± 82 fs for the ML models at scaling factors 15, 25 and 35, respectively. These time constants are used to extrapolate to α = 1 for both MR-CISD (see Figure 4b) and ML (see Figure 4c). For the Fit1 method, the MR-CISD gives time constant of 434 ± 106 ps, while ML gives 316 ± 67 ps. For the Fit2 method, MR-CISD yields 225 ± 1771 ps and ML gives 218 ± 841 ps. While the extrapolation using Fit2 method agrees closely, this is likely a coincidence resulting from the different values of τ^α^ and the fitted slopes (−1.86 for ML and –1.77 for MR-CISD, both of which are near the theoretical value of –2). The Fit1 method more closely highlights the difference in the data points, as the fixed exponent leads to a more pronounced divergence in the extrapolated values.

The discrepancy in the time constants is mainly caused by the ensemble of trajectories corresponding to scaling factor 35. This is evident if we replace the ML population with the MR-CISD reference, effectively assuming the trajectories at α = 35 were perfectly reproduced by the ML models (see Figure S6a). In such a scenario, the recomputed ML time constants would be 1762 ± 369, 742 ± 154, and 407 ± 85 fs, leading to extrapolated time constants of 407 ± 68 and 190 ± 420 ps for the Fit1 and Fit2 methods (see Figure S6b), respectively, which are closer to the reference values of 434 ± 106 and 225 ± 1771 ps. One possible interpretation is that ML errors are amplified by the scaling factor, leading to noisier and more strongly fluctuating population transfer for larger values of α, an effect that could be mitigated by an AL framework. This observation also argues against using ML to increase the number of trajectory ensembles *N* by adding points with large scaling factors α. Given the evidence, we recommend using a hybrid approach: running one set of trajectories with the largest scaling factor, then using the resulting data to train ML models that are subsequently employed with smaller scaling factors. This approach would benefit from MR-CISD data within multicurve fitting, while the ML models would save a substantial amount of time. However, the current results suggest that ML models should be applied with special care within an accelerated SH scheme, given the high sensitivity of the extrapolated results to the fitted time constants.

The reported time constants have large bootstrapping errors, which are amplified during the extrapolation. While the fitting errors might be reduced by propagating longer trajectories, this may not be feasible for costly QM methods. Moreover, ML models can be difficult to train to cover a larger configurational space, and extrapolation outside the training domain may introduce additional errors. Fortunately, ML makes it possible to run much larger trajectory ensembles. In our case, we performed an additional 300 trajectories, increasing the total number to 500 (see Figures 5 and S2; same applies to Figures S14–S15), although significantly larger ensembles should become feasible once the entire procedure is optimized. For the larger ensemble, the bootstrapping errors are substantially reduced, yielding time constants of 928 ± 173, 363 ± 53, and 199 ± 30 fs, and extrapolated time constants of 212 ± 25 and 127 ± 170 ps for the Fit1 and Fit2 methods, respectively. Although these results cannot be directly compared with the MR-CISD reference, they demonstrate that ML can substantially improve the reliability of the accelerated SH method by enabling much larger trajectory ensembles.

**Figure 5:**
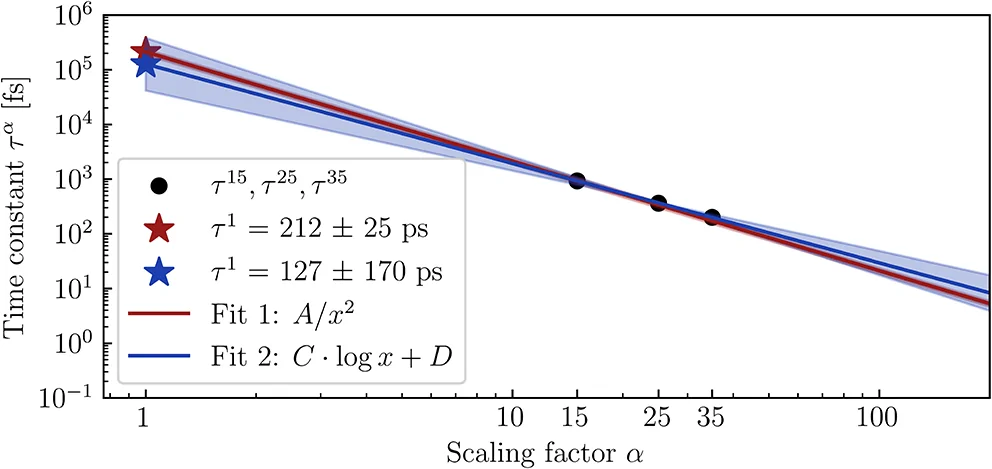
Extrapolation of ML time constants using Fit1 and Fit2 methods to scaling factor α = 1 obtained from 500 ML trajectories with scaling factors 15, 25, and 35. The confidence intervals for the extrapolated values are obtained by applying the same extrapolation formula using τ ± the 5000-sample bootstrap error.

## Conclusions

In this work, we investigated the reliability of accelerated SH scheme, which relies on the scaling of SOCs and subsequent extrapolation to determine long-range time constants. Using both Fit1 and Fit2 methods, we demonstrated that small trajectory ensembles are insufficient for providing reliable results. The Fit1 approach relies on a fixed theoretical scaling of α^–2^, whereas the Fit2 approach utilizes a log–log transformation to fit the scaling exponent as a free parameter. The latter is preferred as it does not restrict the exponent to −2, a constraint strictly applicable only to two-state systems, although our fits yielded values close to this theoretical value. However, the additional fitting parameter increases the bootstrapping errors, an effect that can be mitigated by using a sufficiently large trajectory ensemble. We recommend employing a multicurve fitting strategy, which ensures stable extrapolation and yields physically reasonable slopes. For the specific case of silaethylene, our analysis indicates that at least 100 trajectories per ensemble are necessary to achieve converged time constants and an even larger number of trajectories is required to reduce the bootstrapping errors.

Furthermore, we explored the integration of ML to enhance reliability of accelerated SH scheme. We trained an MS-ANI model for the singlet PESs and separate ANI model for the triplet state. The RPR procedure, combined with the gradient difference descriptor and a phase-correction algorithm, was used to fit NACs. This approach was extended to learning of SOCs directly from Cartesian coordinates, translated to center of mass and rotated using tensor of inertia. All models achieved satisfactory performance in preliminary static tests.

Our assessment of various ML enhancement opportunities revealed that increasing the number of trajectory ensembles (*N*) does not significantly improve the extrapolation scheme compared to increasing the number of trajectories (*n*) within each ensemble. If the scaling factor is too small, the triplet population growth becomes difficult to fit; conversely, extending the simulation time to compensate for slow growth might lead to instabilities as the ML model extrapolates beyond its training data range. Furthermore, large scaling factors might amplify the inaccuracies in the SOCs as shown for scaling factor 35, negatively influencing the resulting populations.

In conclusion, the most promising application of ML in this context is to expand the size of an ensembles, provided the ML models possess sufficient accuracy. While the *S*_1_ → *S*_0_ relaxation is in excellent agreement and ML-predicted populations generally fall within the 95% confidence interval of the MR-CISD reference, the observed discrepancies in the fitted time constants suggest that special care must be taken when assembling training sets. Future work will focus on the implementation of AL procedures to refine these models and improve the overall robustness of ML-accelerated nonadiabatic dynamics.

## Supplementary Material



## Data Availability

The data underlying this study, the Jupyter notebook used for the analysis, and any additional scripts and code are available from FigShare: 10.6084/m9.figshare.33071360. The machine learning part of this work was done using open-source MLatom under the Apache 2.0 License in https://github.com/dralgroup/mlatom.

## Notes

Extrapolation of ML time constants for different ensemble size; population obtained from 200 MR-CISD and 500 ML trajectories; extrapolation of MR-CISD time constants with 4 ensembles; projection of MR-CISD and ML trajectories to β−θ plane; comparison of MR-CISD and ML results from FSSH simulations of silaethylene; population obtained from 200 ML trajectories with scaling factor 100; histogram of spin−orbit coupling magnitudes for 100,100 points used for assembling training sets; trajectory-averaged S_0_ and T_1_ energies; alternative extrapolation of ML time constants; and projection and histograms of geometries collected from 200 MR-CISD trajectories (PDF)
