# Supplementary material for: Toward Machine Learning Enhancement of Accelerated Surface Hopping with Scaled Spin–Orbit Couplings
Source: J Phys Chem A. 2026 Oct 23;130(44):8997–9008. doi: 10.1021/acs.jpca.6c06078 (PMC13746802; doi:10.1021/acs.jpca.6c06078)
Supplement: Supplementary file 1 [file jp6c06078_si_001.pdf]

Supporting Information: Toward Machine Learning Enhancement of Accelerated Surface Hopping with Scaled Spin–Orbit Couplings

Jakub Martinka,[†,‡] Mahesh Kumar Sit,[†] Pavlo O. Dral,[*,¶,§,‖,⊥] and Jiří Pittner[*,†]

†*Heyrovský Institute, Academy of Sciences of the Czech Republic, v.v.i., Dolejškova 3, 18223 Prague 8, Czech Republic*

‡*Department of Physical and Macromolecular Chemistry, Faculty of Science, Charles University, Hlavova 8, 128 43 Prague 2, Czech Republic*

¶*State Key Laboratory of Physical Chemistry of Solid Surfaces, Department of Chemistry, College of Chemistry and Chemical Engineering, and Fujian Provincial Key Laboratory of Theoretical and Computational Chemistry, Xiamen University, Xiamen 361005, China*

§*Institute of Physics, Faculty of Physics, Astronomy, and Informatics, Nicolaus Copernicus University in Toruń, ul. Grudziadzka 5, 87-100 Toruń, Poland*

‖*Institute of Advanced Studies, Nicolaus Copernicus University in Toruń, ul. Wileńska 4, 87-100 Toruń, Poland*

⊥*Aitomistic, Shenzhen 518000, China*

E-mail: dral@xmu.edu.cn; jiri.pittner@jh-inst.cas.cz

Errors related to the size of an ensemble and the number of ensembles

The accelerated surface hopping approach is based on artificially scaling the couplings driving the process by a constant $\alpha > 1$. Then, N ensembles of n trajectories (typically $N = 3$ and n depends on the level of theory and the molecular system of interest) are obtained by performing accelerated surface hopping with different α values. The resulting populations are fitted to obtain the time constants τ^{α}, which are subsequently used for extrapolation to obtain $\tau^{\alpha=1}$. The requirement to generate several ensembles increases the computational cost, and the number of trajectories within each ensemble is typically sacrificed. Machine learning reduces the computational demand while maintaining the accuracy of reference quantum chemical method. There are several opportunities for ML to enhance the reliability of accelerated surface hopping approach. One of the possibilities is to increase n, the number of trajectories in the ensembles that are used for the extrapolation. We identify this possibility as the most important due to its effect of reducing errors related to the fitted time constants $\{\tau^{\alpha}\}$, which directly influence the extrapolation to $\tau^{\alpha=1}$. The effect of the n is demonstrated in the main text in Fig. 2 by showing the dependence of $\tau^{\alpha=1}$ on n and that at least 100 trajectories is needed to obtain reliable fits. The same trend is observed in case of ML trajectories (see Fig. S1).

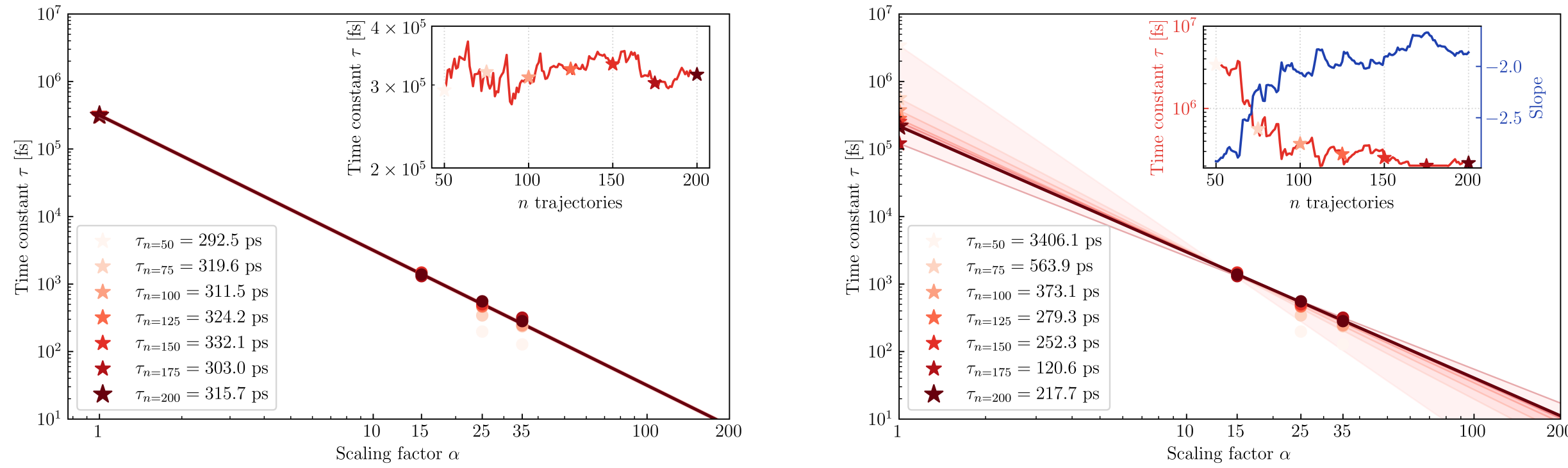

(a) `Fit1` method.

(b) `Fit2` method.

Figure S1: Extrapolation of ML time constants to $\alpha = 1$ using `Fit1` and `Fit2` methods, based on the number of trajectories n. The top-right plot shows the time constants obtained by analyzing n trajectories; in the case of `Fit2`, the slope is shown in blue. Scaling factors of 15, 25, and 35 were used.

Furthermore, a large n reduces the error interval obtained by the bootstrapping procedure as can be seen in the case of 500 trajectories in Fig. S6 (compared with Fig. 4a) and in Fig. 5 in the main text (compared with Fig. 4c).

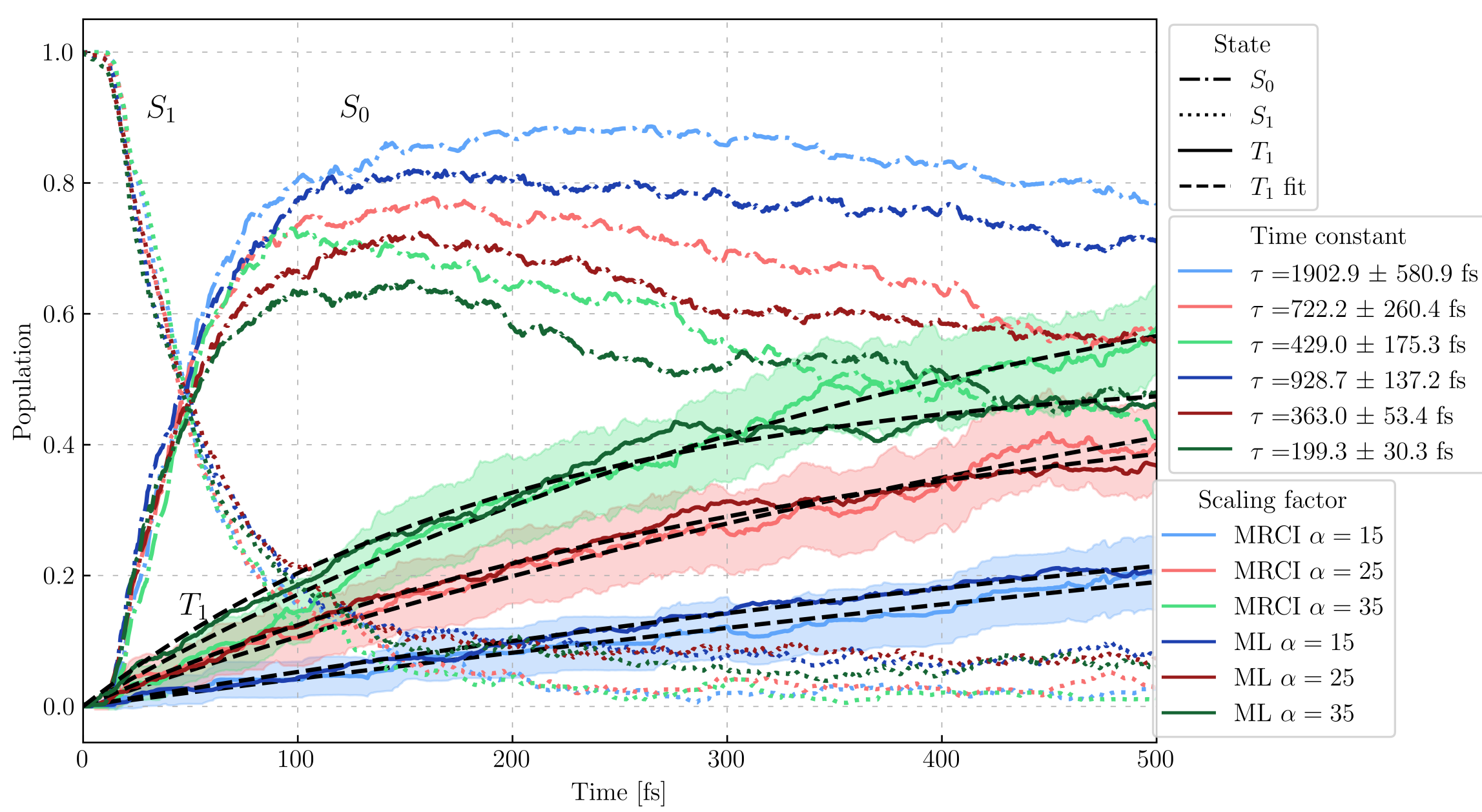

Figure S2: Population obtained from 200 MR-CISD and 500 ML trajectories with scaling factors 15, 25, and 35. The standard errors are estimated using a 5000-sample bootstrap procedure.

Another possibility is to increase N, the number of ensembles used for the extrapolation. We observe that increasing N within the existing range of scaling factors, e.g. by using $\{15, 20, 25, 35\}$ (see Fig. S3) instead of $\{15, 25, 35\}$ (see Fig. 2 in the main text), does not significantly affect the resulting time constant compared with the uncertainty related to a small n.

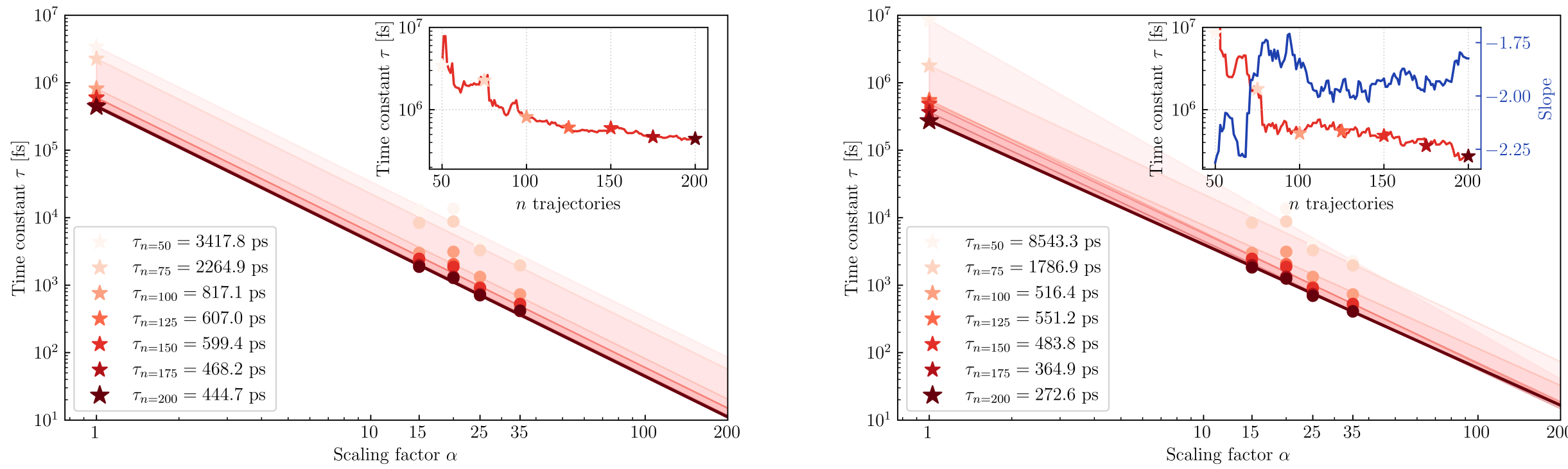

(a) `Fit1` method. (b) `Fit2` method.

Figure S3: Extrapolation of MR-CISD time constants to $\alpha = 1$ using `Fit1` and `Fit2` methods, based on the number of trajectories n. The top-right plot shows the time constants obtained by analyzing n trajectories; in the case of `Fit2`, the slope is shown in blue. Scaling factors of 15, 20, 25, and 35 were used.

Projections to the $\beta - \theta$ plane

The accuracy of ML models is measured on the test sets, but more relevant is the performance of the ML models in production-level simulations. In the case of silaethylene, the initial stage of the dynamics is crucial for further evolution due to the two types of trajectories. Type T trajectories are characterized by simultaneous torsional and Si–C stretching. The latter lifts the degeneracy, causing the trajectories to hop later compared with the type B trajectories, which are driven by the Si–C stretch into the region of the bipyramidalized crossing seam. Type B trajectories reach the crossing seam faster than type T trajectories, meaning that the ratio of the two modes must be reproduced by the ML models to achieve agreement with the reference S_1 population decay. Type B and type T trajectories can be characterized by

additional coordinates, θ and β, defined as

$$\theta = \sqrt{\frac{\theta_1^2 + \theta_2^2}{2}}, \tag{1a}$$

$$\beta = \sqrt{\frac{\beta_1^2 + \beta_2^2}{2}}, \tag{1b}$$

where the dihedral angles θ_1 (H_1-Si-C-H_3) and θ_2 (H_2-Si-C-H_4) capture the central torsion, whereas β_1 (C-H_1-H_2-Si) and β_2 (Si-H_3-H_4-C) describe the relative orientation of the methylene and silylene groups. The projection of MR-CISD and ML trajectories onto the $\beta - \theta$ plane (type T and type B are separated by the $\beta = 0.95\theta$ line) provides an ideal test for the ML-PESs. This projection is presented in the main text in Fig. 3 for $\alpha = 15$, but α does not influence this projection in either case, as can be seen in Fig. S4 and Fig. S5.

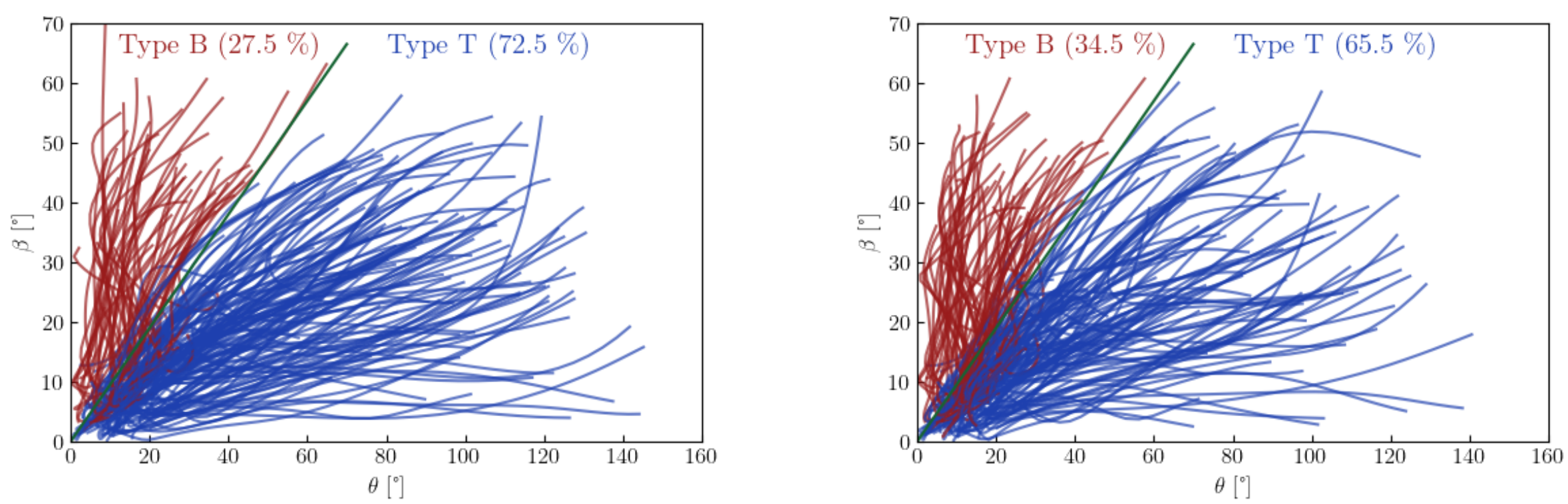

Figure S4: Projection of MR-CISD (left) and ML (right) trajectories with scaling factor $\alpha = 25$ onto $\beta - \theta$ plane up to 20 fs. The deactivation process of silaethylene is well recovered by ML-driven trajectories.

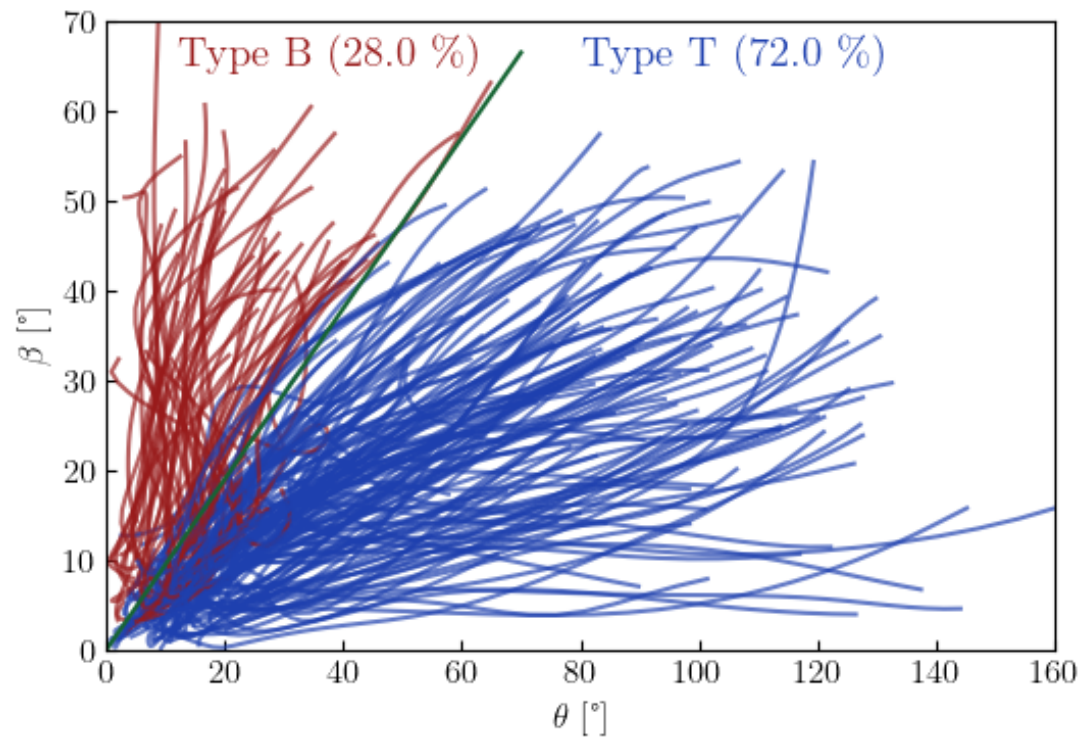

Figure S5: Projection of MR-CISD (left) and ML (right) trajectories with scaling factor $\alpha = 35$ onto $\beta - \theta$ plane up to 20 fs. The deactivation process of silaethylene is well recovered by ML-driven trajectories.

Population fits with identical T_1 populations for $\alpha = 35$, without multi-curve fitting and for $\alpha = 100$

The discrepancy in the time constants extrapolated from 200 MR-CISD and 200 ML ensembles of $\alpha = \{15, 25, 35\}$ (see Fig. 4b and Fig. 4c in the main text) is mainly caused by the ensemble of trajectories corresponding to the scaling factor 35. This is evident if we replace the ML population with the MR-CISD reference, effectively assuming that the trajectories at $\alpha = 35$ were perfectly reproduced by the ML models (see Fig. S6a). In such a scenario, the recomputed ML time constants would be 1762, 742, and 407 fs, leading to extrapolated time constants of 407 ps and 190 ps for the `Fit1` and `Fit2` methods (see Fig. S6b), respectively, which is closer to the reference values of 434 ps and 225 ps (see Fig. 4b). Despite having the same population in the case of $\alpha = 35$, the $\tau^{\alpha=35}$ values of MR-CISD and ML trajectories differ. This is a consequence of the multi-curve fitting approach, which is based on sharing the A^{pop} constant, which stabilizes the extrapolation scheme. Once the populations are fitted independently, the extrapolation scheme tends to be unstable, as can be seen from Fig. S7a and Fig. S7b. Therefore, the MR-CISD and ML time constants differ unless exactly the same triples are employed for the fitting.

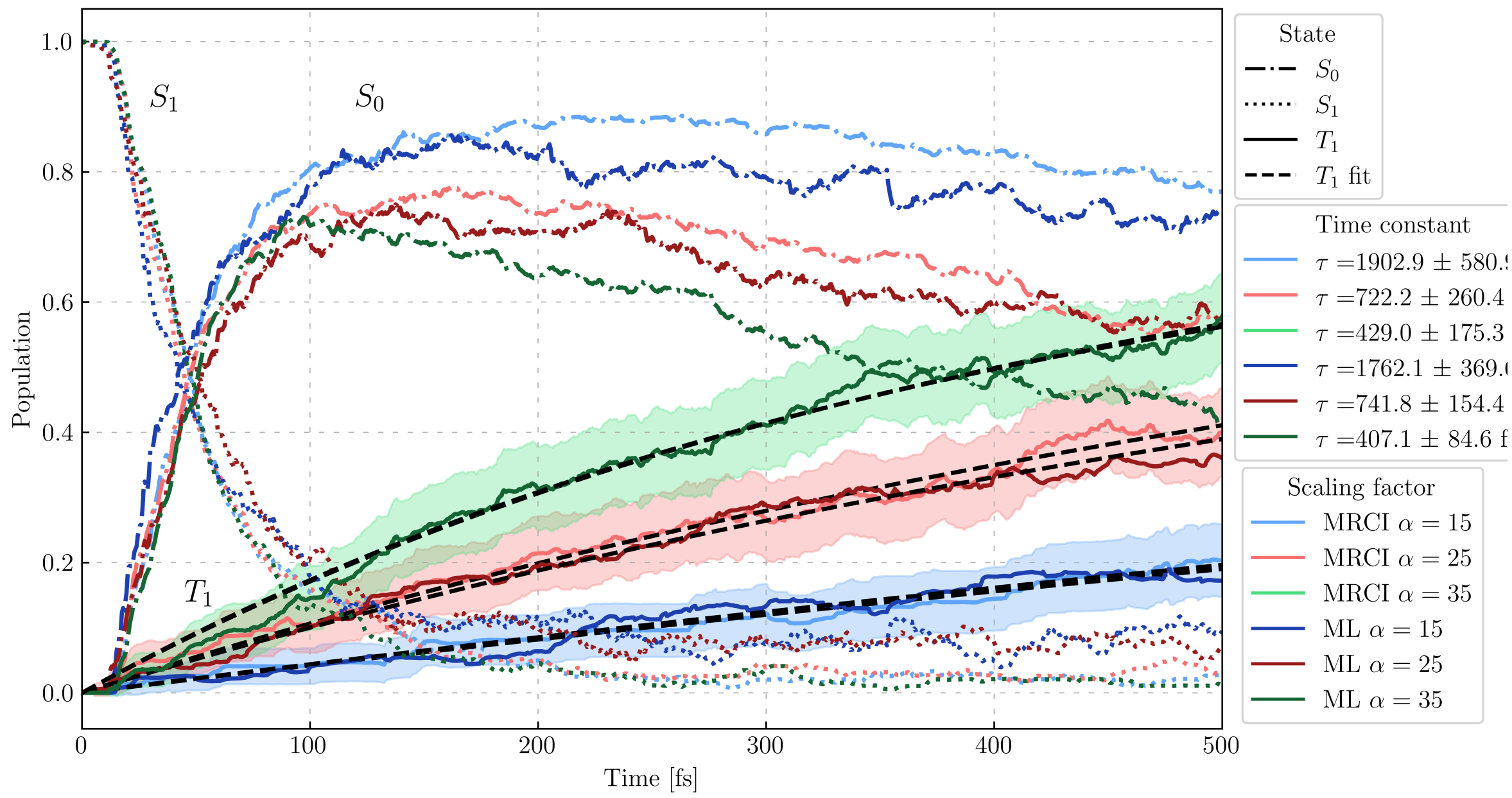

(a) Populations obtained from 200 MR-CISD and 200 ML trajectories with scaling factors of 15, 25, and 35. The ML T_1 population for $\alpha = 35$ is replaced by the MR-CISD population for fitting.

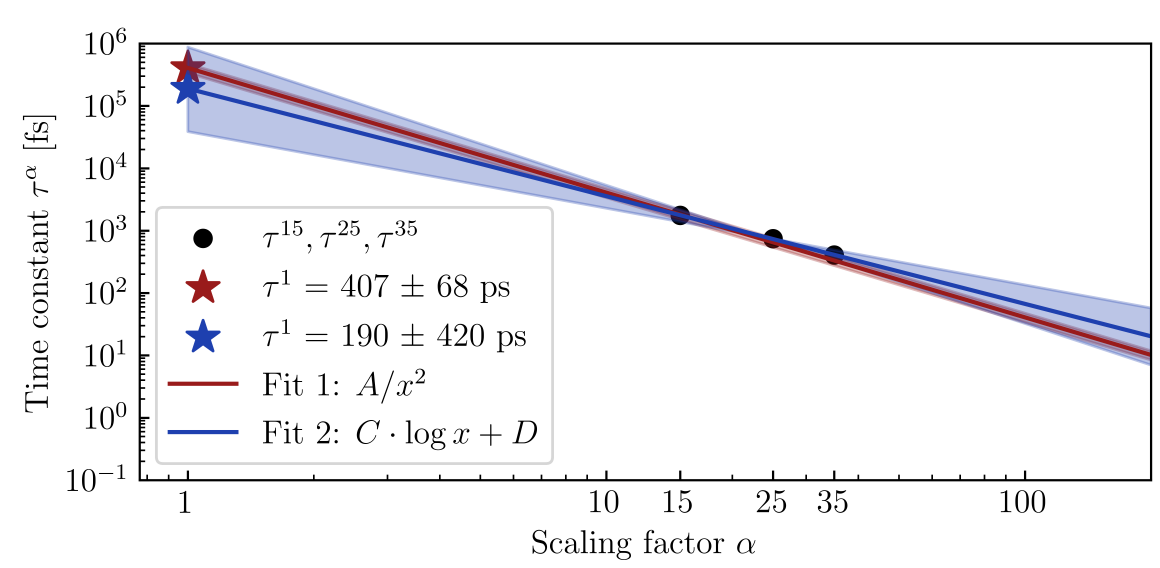

(b) Extrapolation of ML time constants using Fit1 and Fit1 methods to scaling factor $\alpha = 1$.

Figure S6: Comparison of MR-CISD and ML results from FSSH simulations of silaethylene, if trajectories at $\alpha = 35$ were perfectly reproduced by the ML models. The standard errors are estimated using a 5000-sample bootstrap procedure, while the confidence intervals for the extrapolated values are obtained by applying the same extrapolation formula using $\tau \pm$ the bootstrap error.

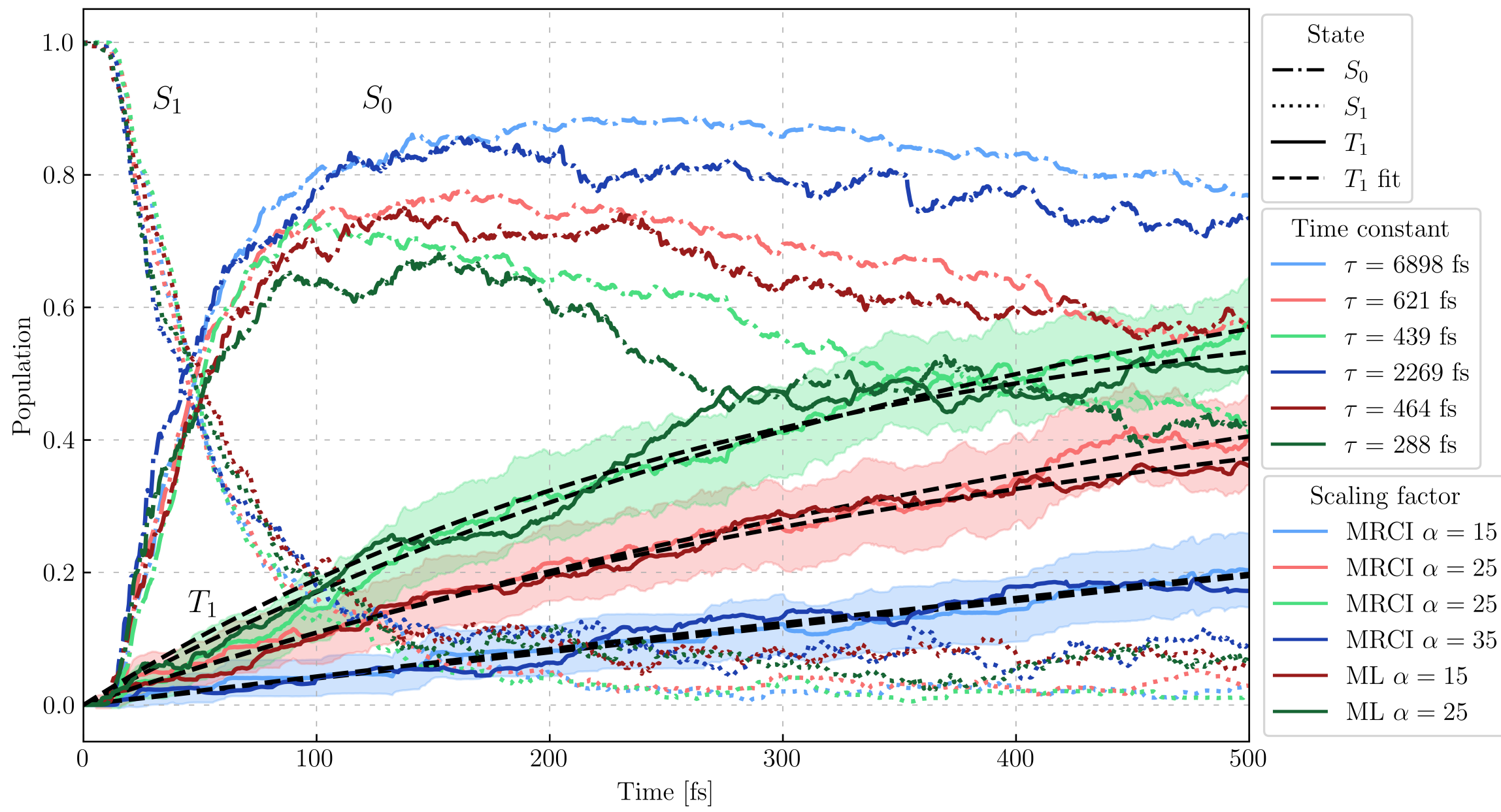

(a) Populations obtained from 200 MR-CISD and 200 ML trajectories with scaling factors of 15, 25, and 35. The τ values were obtained without using a multi-curve fitting.

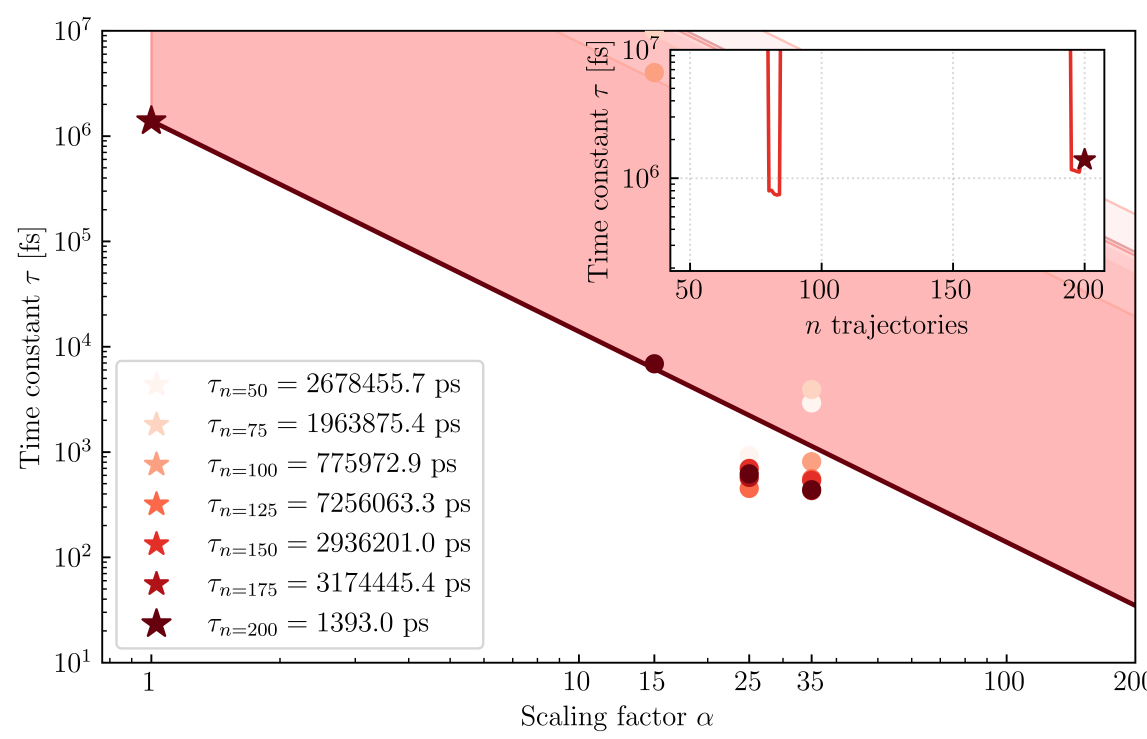

(b) Extrapolation of ML time constants using the `Fit1` and `Fit2` methods to a scaling factor of $\alpha = 1$.

Figure S7: Comparison of MR-CISD and ML results from FSSH simulations of silaethylene without using multi-curve fitting. The extrapolated values are unstable because the multi-curve fitting approach was not employed.

To fit the triplet populations, we use $A^{\text{pop}}(1 - \exp(-t/\tau_\alpha))$, which contains A^{pop} corresponding to the asymptotic maximum triplet population. Using small values of α leads to relatively slow growth of the triplet population, which can be seen in Fig. S2. As can be seen from Fig. S8, the equilibrium between S_1 and T_1 is achieved within 100 fs when $\alpha = 100$,

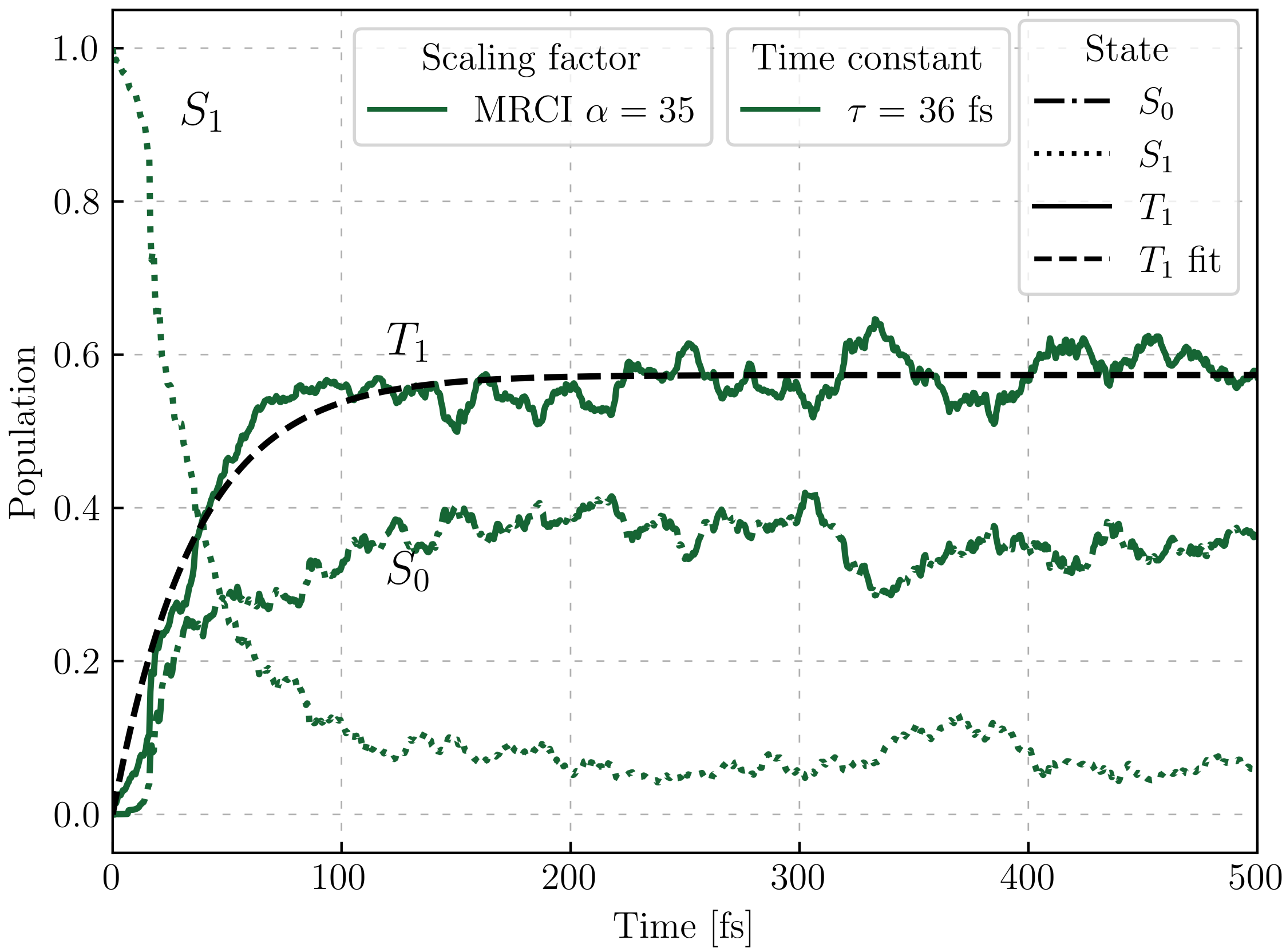

Figure S8: Population obtained from 200 ML trajectories with scaling factor 100.

which justifies the use of A^{pop} as a fitting parameter.

Spin–orbit coupling magnitudes

The training and test sets were constructed from 100 MR-CISD trajectories with $\alpha = 20$. Each trajectory finished at a final time of 500 fs with a time step of 0.5 fs, which means that a total of 100,100 points were collected. The use of the accelerated surface hopping scheme is justified due to the magnitude of spin–orbit couplings that are always below 70 cm^{-1}, as shown in Fig. S9.

The accessibility of the high-SOC region can be understood from a scatter density plot of the S_0–T_1 energy gap against the unscaled SOC magnitude for MR-CISD trajectories with a scaling factor of 15, which shows the density of visited configurations. Fig. S10 shows that

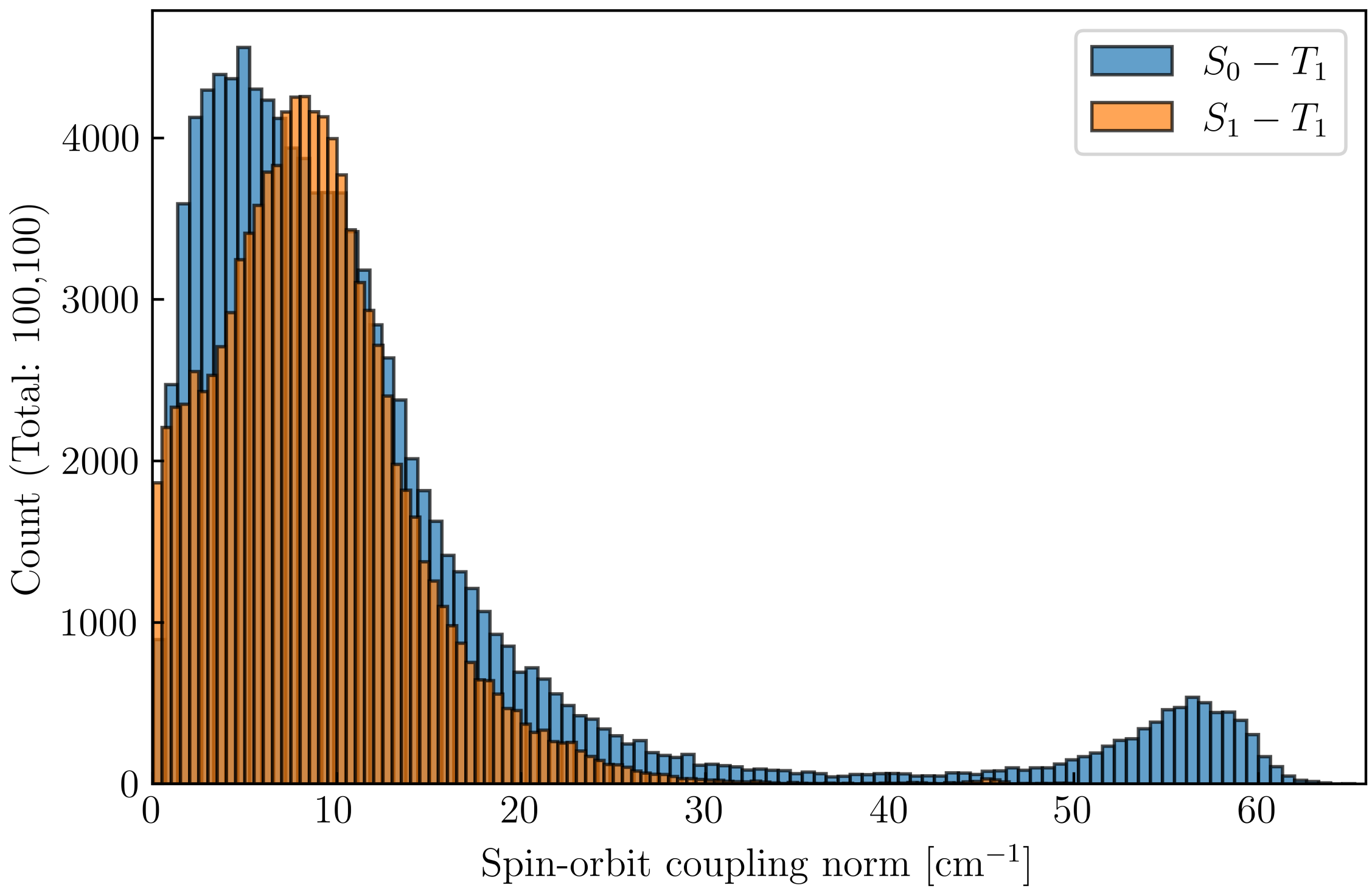

Figure S9: Histogram of spin–orbit coupling magnitudes for 100,100 points used for assembling training sets.

configurations with a small S_0–T_1 gap are populated, but only sparsely populated with a large SOC norm, which justifies the SOC scaling and the slow growth of the T_1 population. Access to the high-SOC region is also linked to overcoming an energy barrier, as shown in Fig. S11, which presents the trajectory-averaged S_0 and T_1 energies, relative to each trajectory's S_0 minimum, as a function of SOC magnitude. The shaded band shows the interquartile range (25th–75th percentile), which overlaps for large SOC values, indicating that ISC becomes accessible once the two states approach near-degeneracy.

Extrapolation with τ_∞ parameter

Instead of A/x^2, $A/x^2 + \tau_\infty$ might be used. While the latter expression is physically motivated, larger errors are reported due to the additional fitting constant for the 500 ML trajectories (compare Fig. S12 and Fig. 5 in the main text).

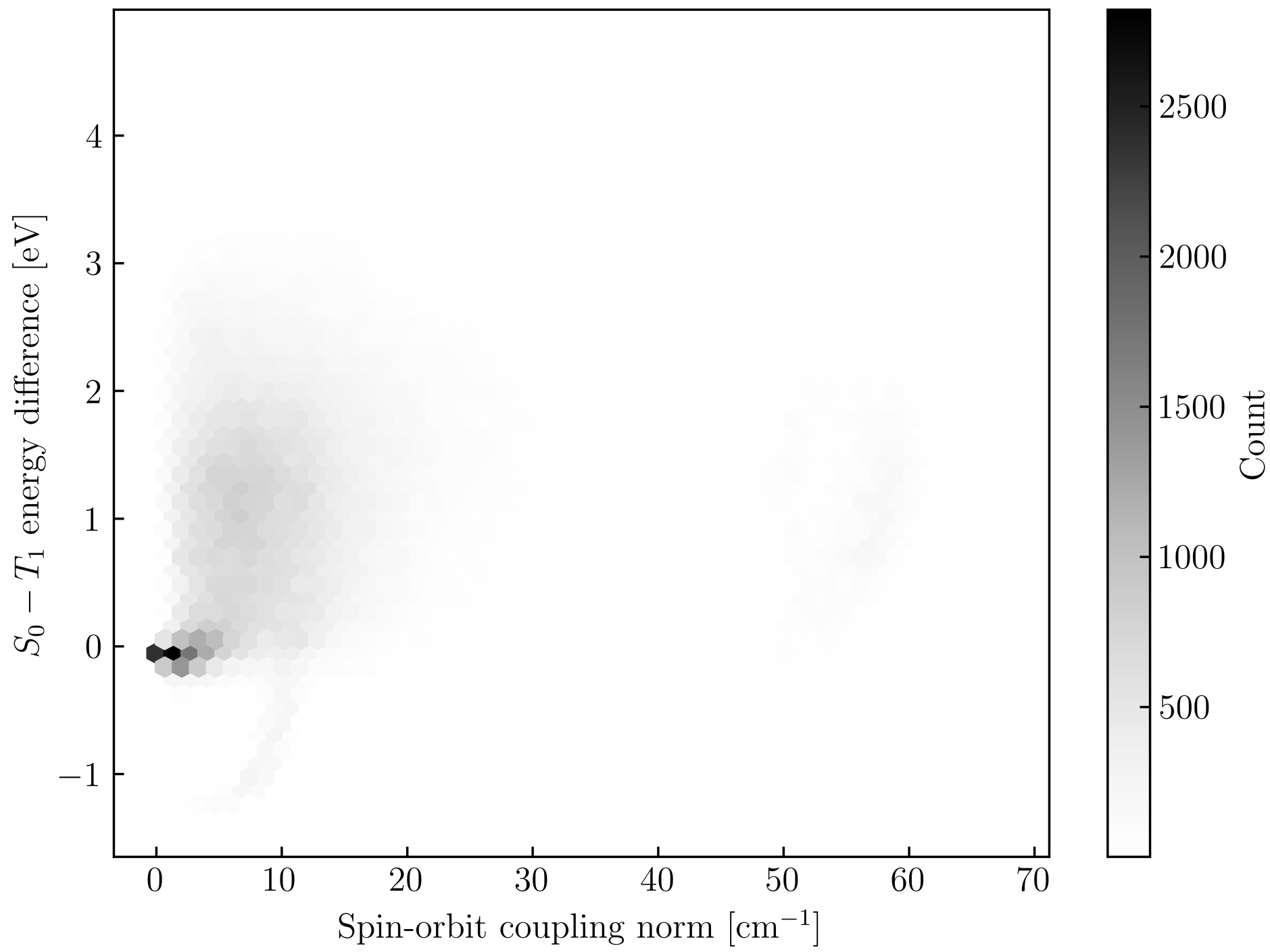

Figure S10: Density of visited configurations in the S_0–T_1 energy gap and unscaled SOC magnitudes over all MR-CISD trajectories with scaling factor 15. The region combining a small energy gap with a large SOC norm is sparsely sampled.

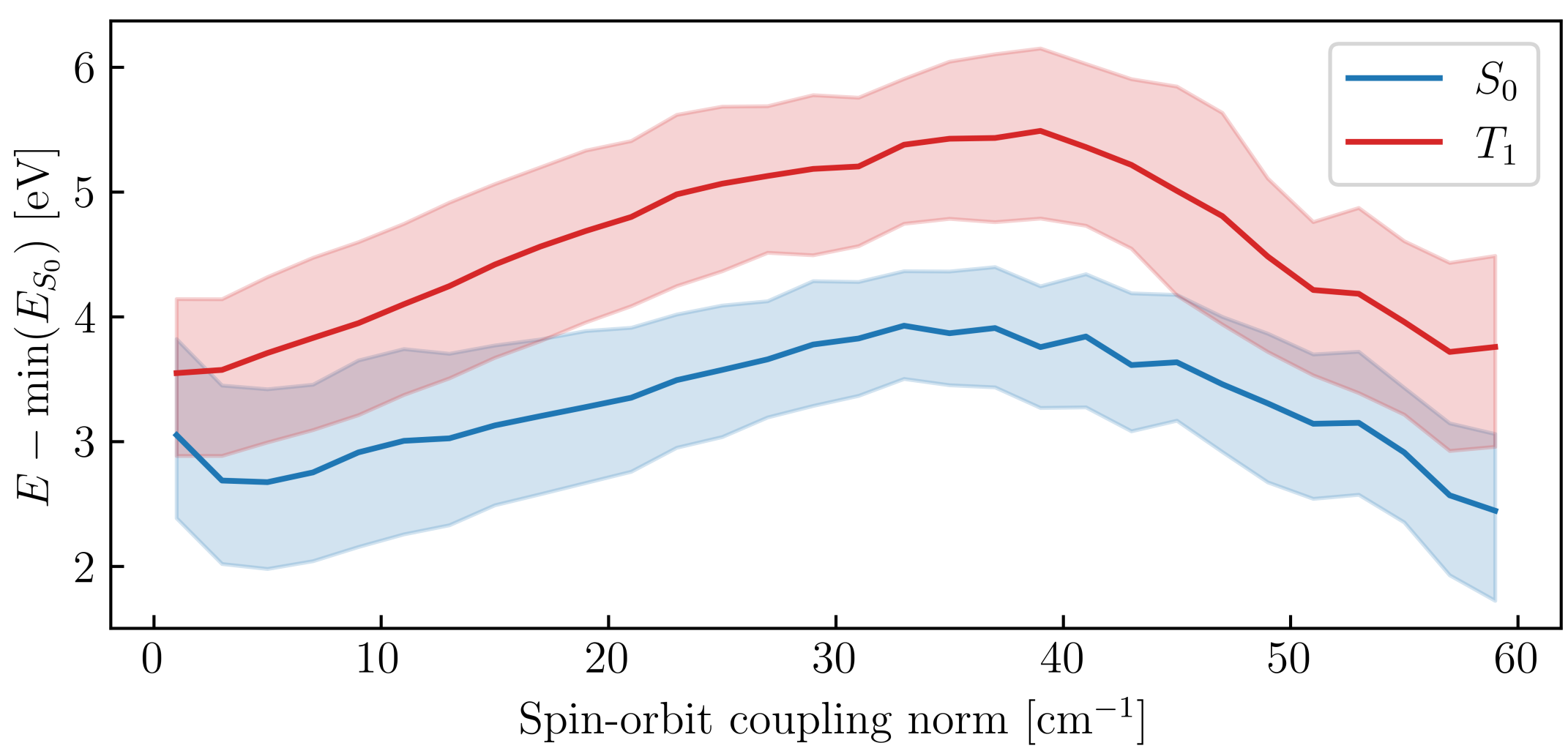

Figure S11: Trajectory-averaged S_0 and T_1 energies, relative to each trajectory's S_0 minimum, as a function of SOC magnitude. Shaded bands denote the interquartile range (25th–75th percentile) across trajectories. The overlap of the two bands at large SOC values reflects the near-degeneracy required for efficient ISC.

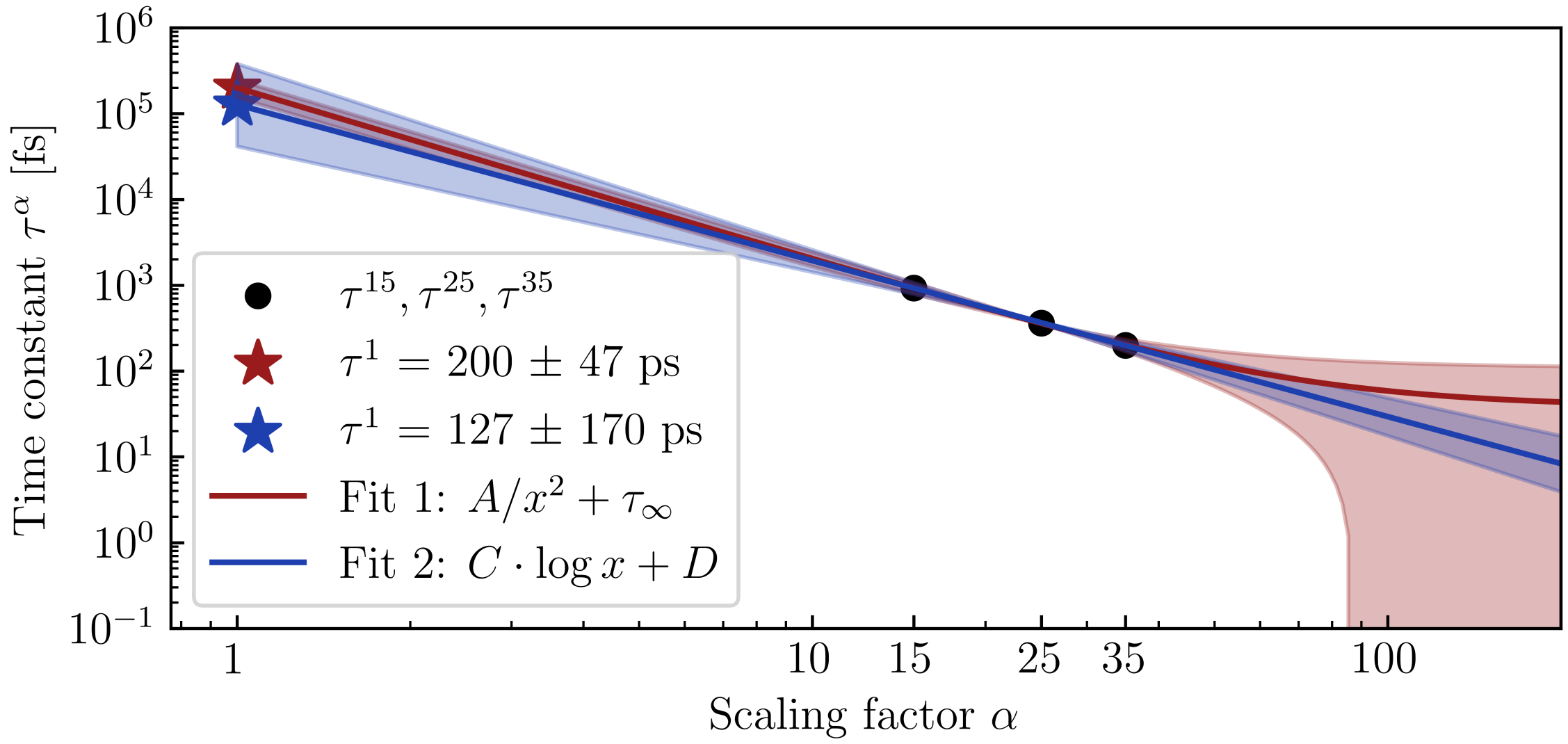

Figure S12: Extrapolation of ML time constants (500 trajectories) using $A/x^2 + \tau_\infty$ and Fit2 methods to scaling factor $\alpha = 1$. The standard errors are estimated using a 5000-sample bootstrap procedure, while the confidence intervals for the extrapolated values are obtained by applying the same extrapolation formula using $\tau\pm$ the bootstrap error.

and for 200 MR-CISD trajectories (compare Fig. S13 and Fig. 4b)

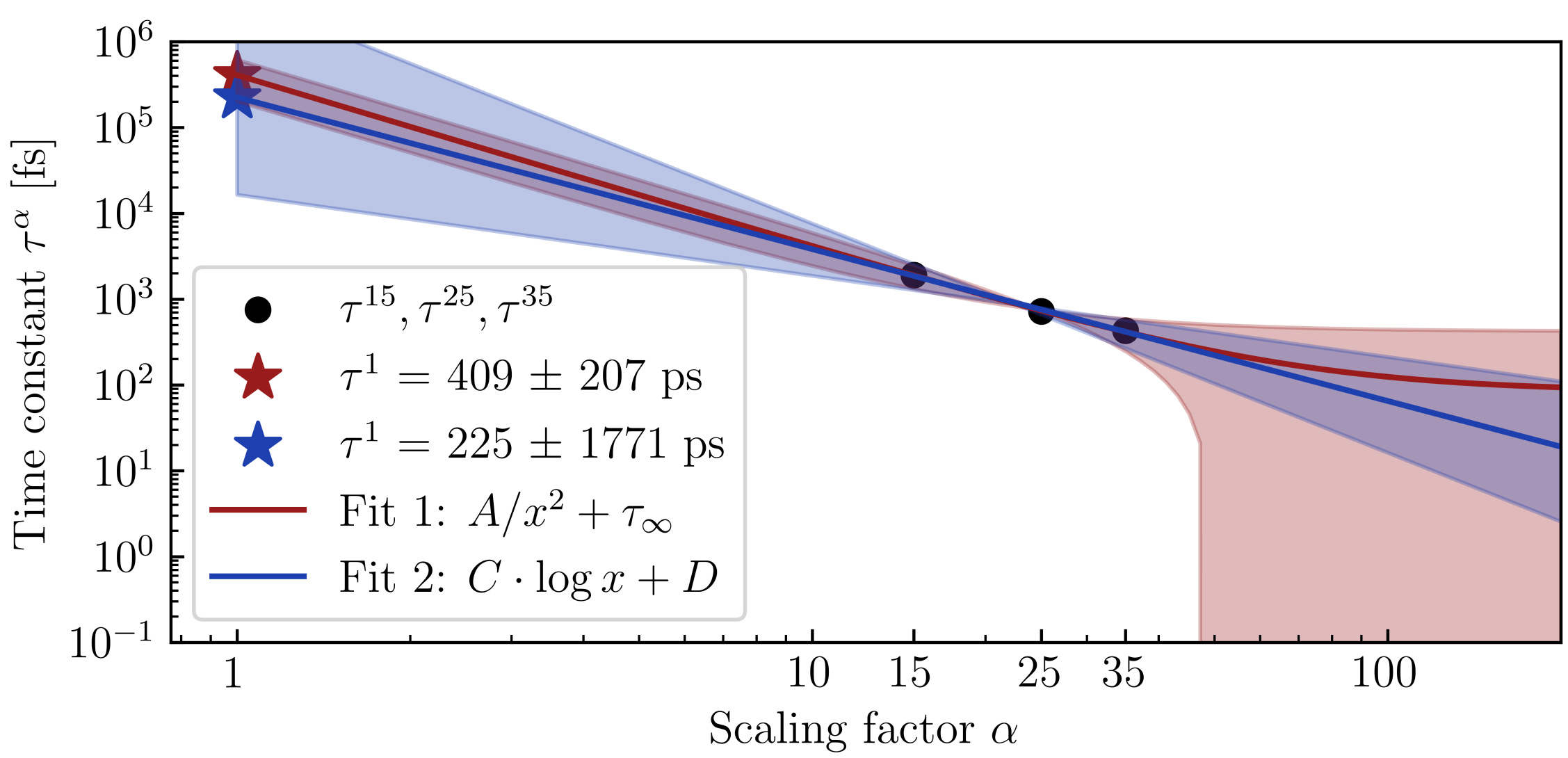

Figure S13: Extrapolation of MR-CISD time constants (200 trajectories) using $A/x^2 + \tau_\infty$ and `Fit2` methods to scaling factor $\alpha = 1$. The standard errors are estimated using a 5000-sample bootstrap procedure, while the confidence intervals for the extrapolated values are obtained by applying the same extrapolation formula using $\tau\pm$ the bootstrap error.

Extrapolation with different initial conditions for α values of 15, 25 and 35

The initial conditions might be biased, but using different initial conditions for each ensemble with different α values leads to comparable results, as can be seen in the case of 500 ML trajectories from a comparison of Fig. S14 and Fig. S2.

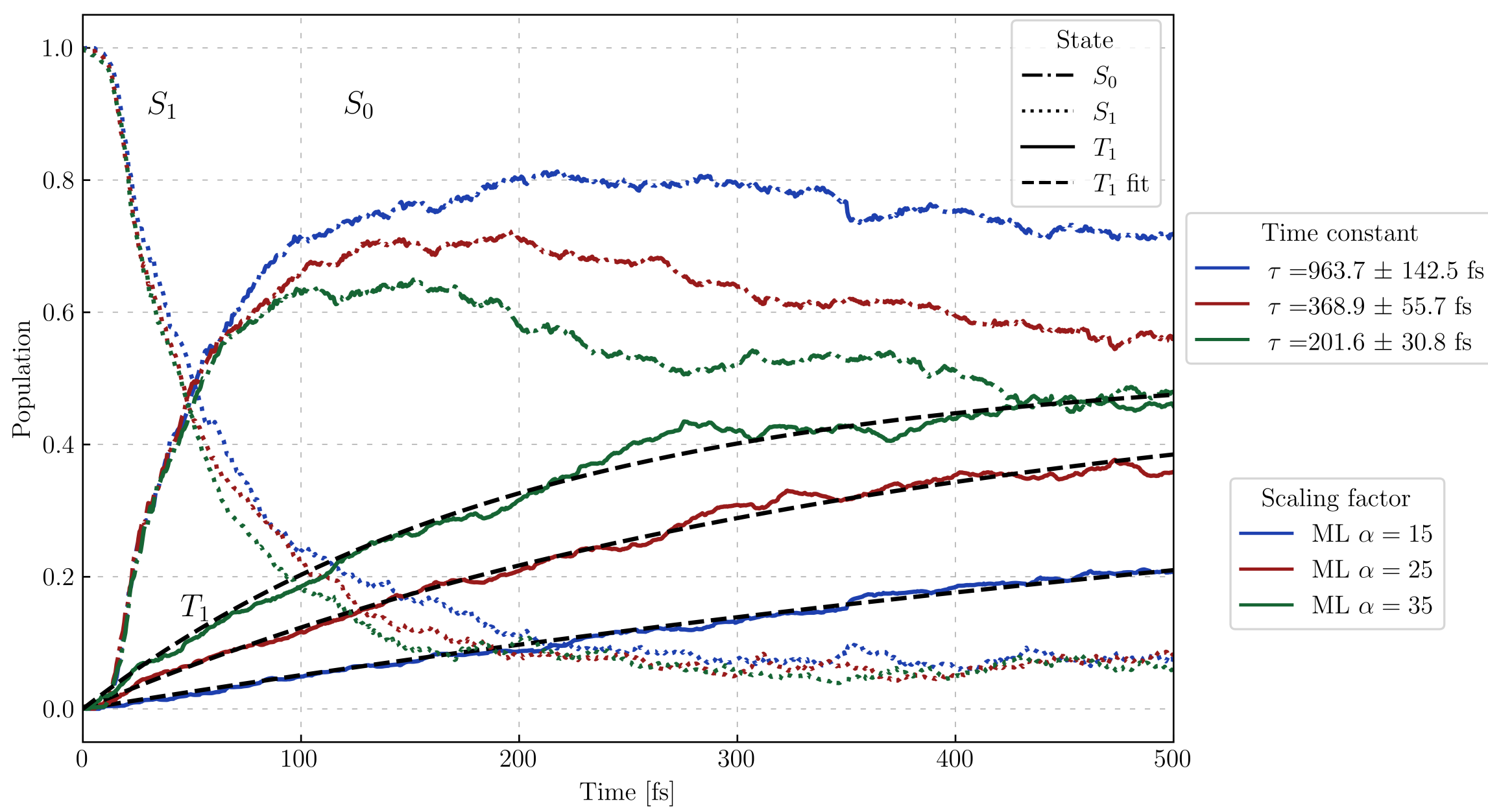

(a) Populations obtained from 500 ML trajectories with diferent initial conditions for each ensemble with the scaling factors of 15, 25, and 35.

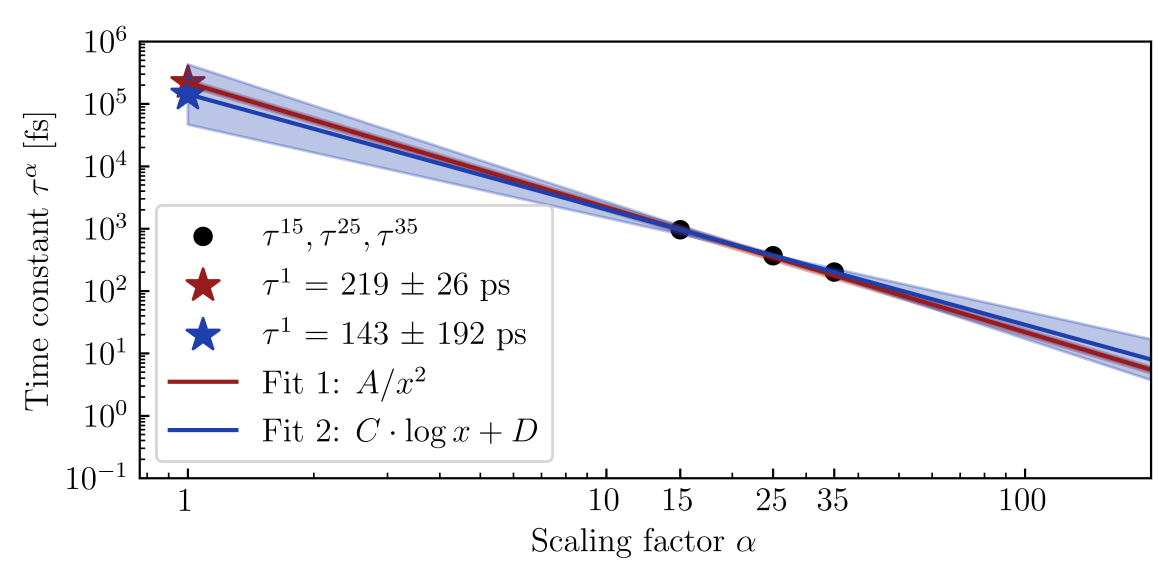

(b) Extrapolation of ML time constants using `Fit1` and `Fit2` methods to scaling factor $\alpha = 1$.

Figure S14: The ML results from FSSH simulations of silaethylene using 500 trajectories per ensemble using different initial conditions. The standard errors are estimated using a 5000-sample bootstrap procedure, while the confidence intervals for the extrapolated values are obtained by applying the same extrapolation formula using $\tau\pm$ the bootstrap error.

Ensembles with small α are more difficult to fit and therefore lead to larger errors. One strategy to mitigate such effects is to use more initial conditions for smaller values of α. We have used 700, 600, and 500 different initial conditions for ML trajectories and conclude that once a sufficiently large number of initial conditions is employed, this uncertainty is mitigated and comparable results are obtained.

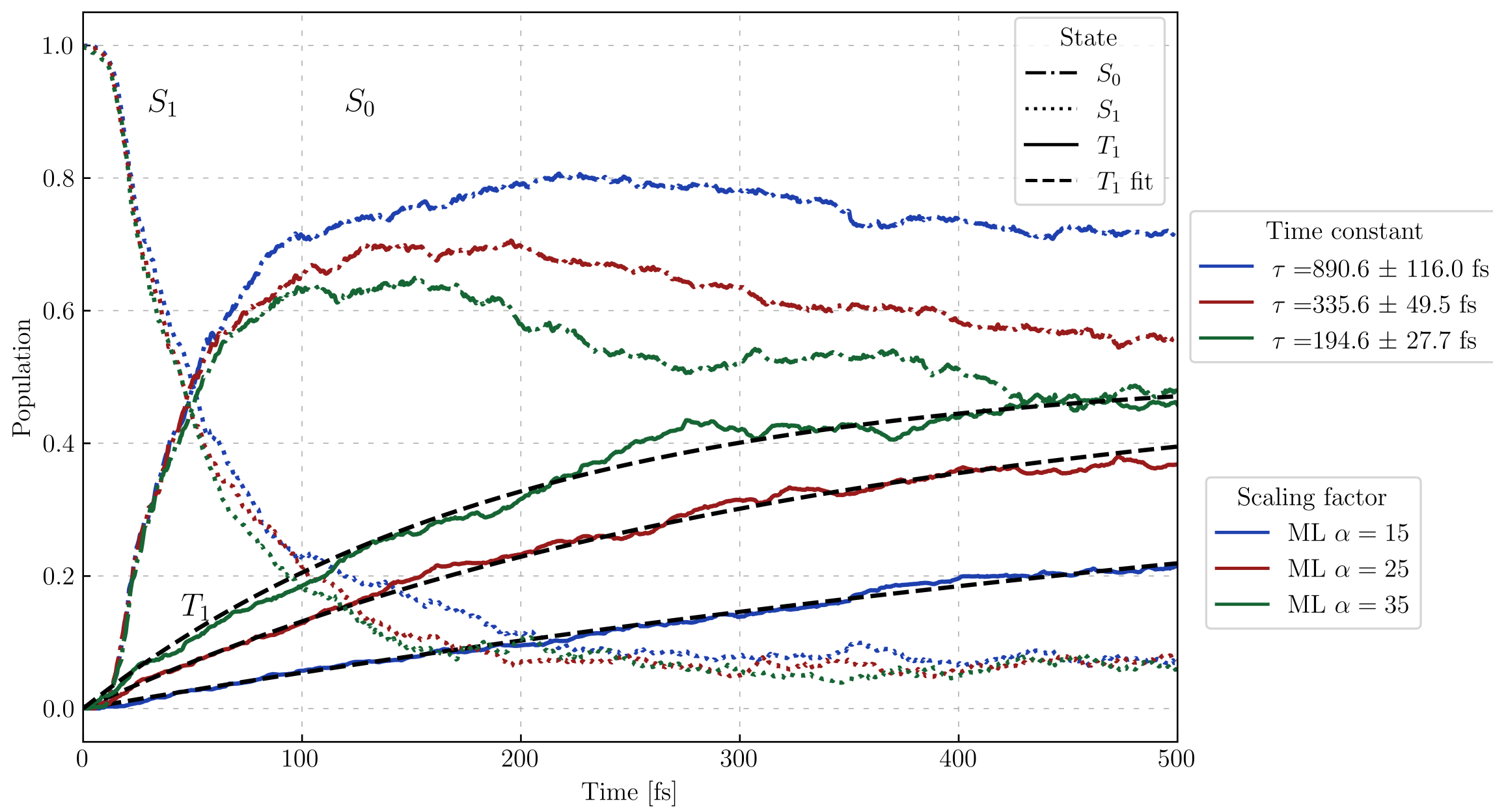

(a) Populations obtained from 700, 600 and 500 ML trajectories for scaling factors of 15, 25, and 35 respectively.

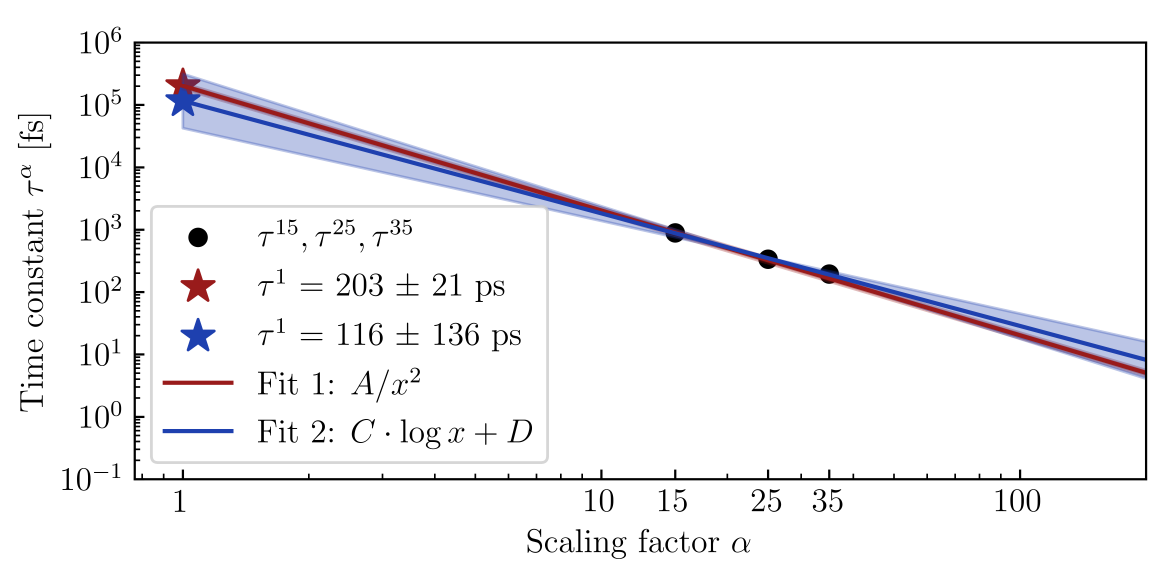

(b) Extrapolation of ML time constants using `Fit1` and `Fit2` methods to scaling factor $\alpha = 1$.

Figure S15: The ML results from FSSH simulations of silaethylene using 700, 600 and 500 ML trajectories for scaling factors of 15, 25, and 35 respectively. The standard errors are estimated using a 5000-sample bootstrap procedure, while the confidence intervals for the extrapolated values are obtained by applying the same extrapolation formula using $\tau\pm$ the bootstrap error.

Molecular configurations related to the increase of T_1 MCH population

We have collected geometries related to the increase of $|c_{T_1}|^2$ in the MCH basis (in the range of 0.5 to 0.95). We project such geometries onto the $\beta - \theta$ plane and color the points by the H_2C–SiH_2 angle (Fig. S16 for $\alpha = 15$, Fig. S17 for $\alpha = 25$, Fig. S18 for $\alpha = 35$), by $E_{T_1} - E_{S_0}$ (Fig. S19 for $\alpha = 15$, Fig. S20 for $\alpha = 25$, Fig. S21 for $\alpha = 35$), and by the magnitude of SOCs (Fig. S22 for $\alpha = 15$, Fig. S23 for $\alpha = 25$, Fig. S24 for $\alpha = 35$). Such an analysis reveals that most of such geometries are related to the type T region. The transfer occurs mostly from S_0 to T_1 once those two states are close to each other, which corresponds to an angle of 90° and high $S_0 - T_1$ SOC value.

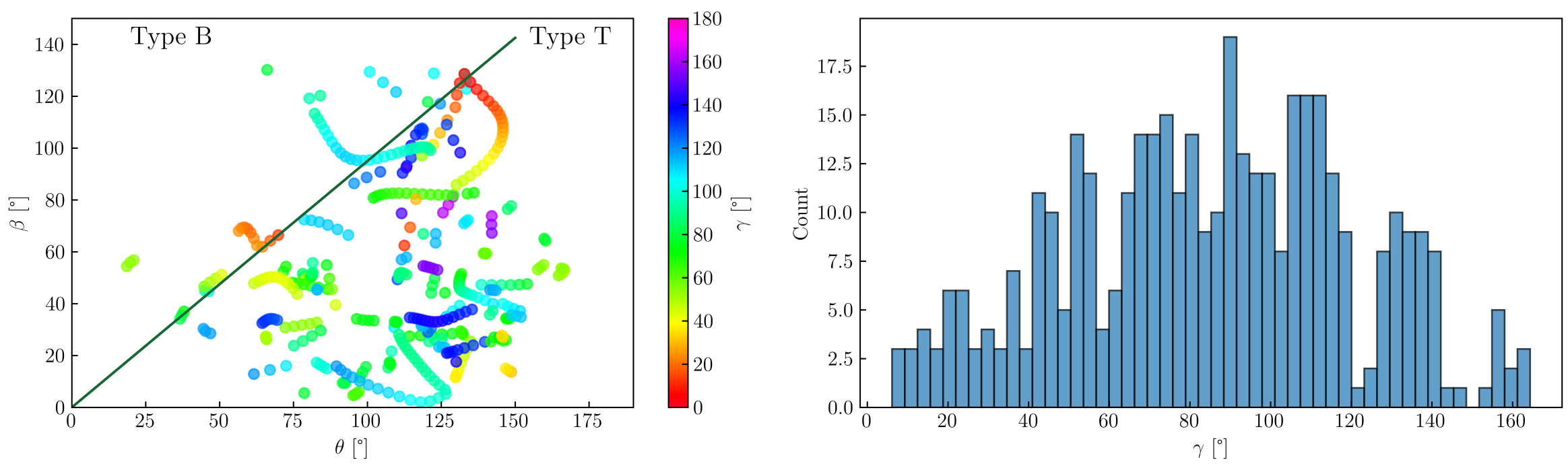

Figure S16: Projection of geometries collected from 200 MR-CISD trajectories with $\alpha = 15$ related to the increase of T_1 population and colored by H_2C–SiH_2 angle (γ).

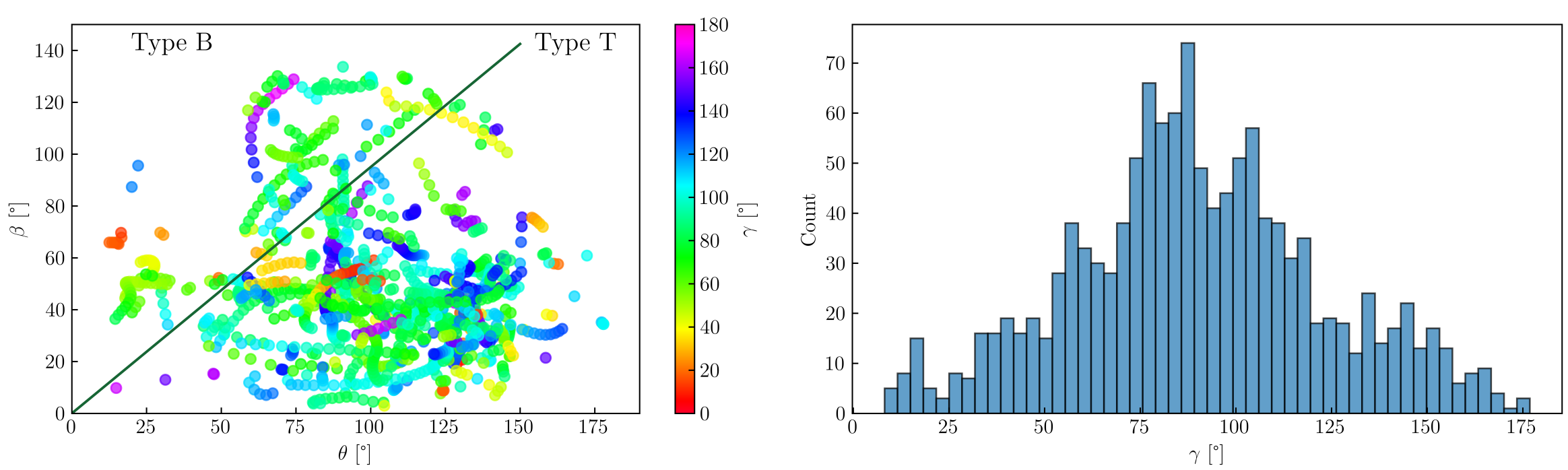

Figure S17: Projection of geometries collected from 200 MR-CISD trajectories with $\alpha = 25$ related to the increase of T_1 population and colored by H_2C–SiH_2 angle (γ).

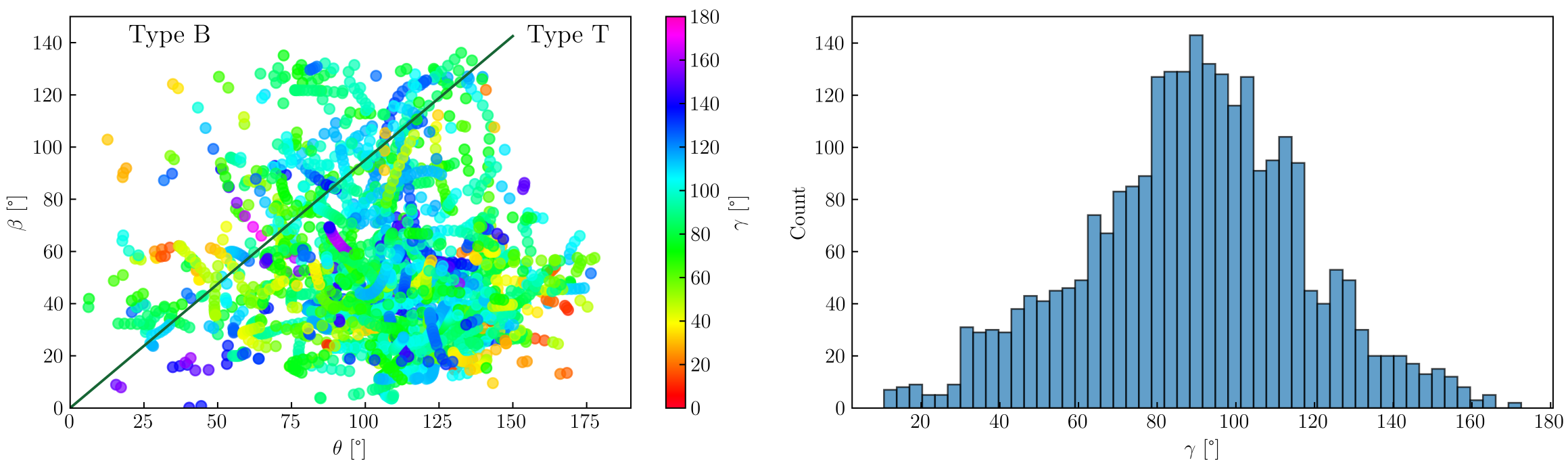

Figure S18: Projection of geometries collected from 200 MR-CISD trajectories with $\alpha = 35$ related to the increase of T_1 population and colored by H_2C–SiH_2 angle (γ).

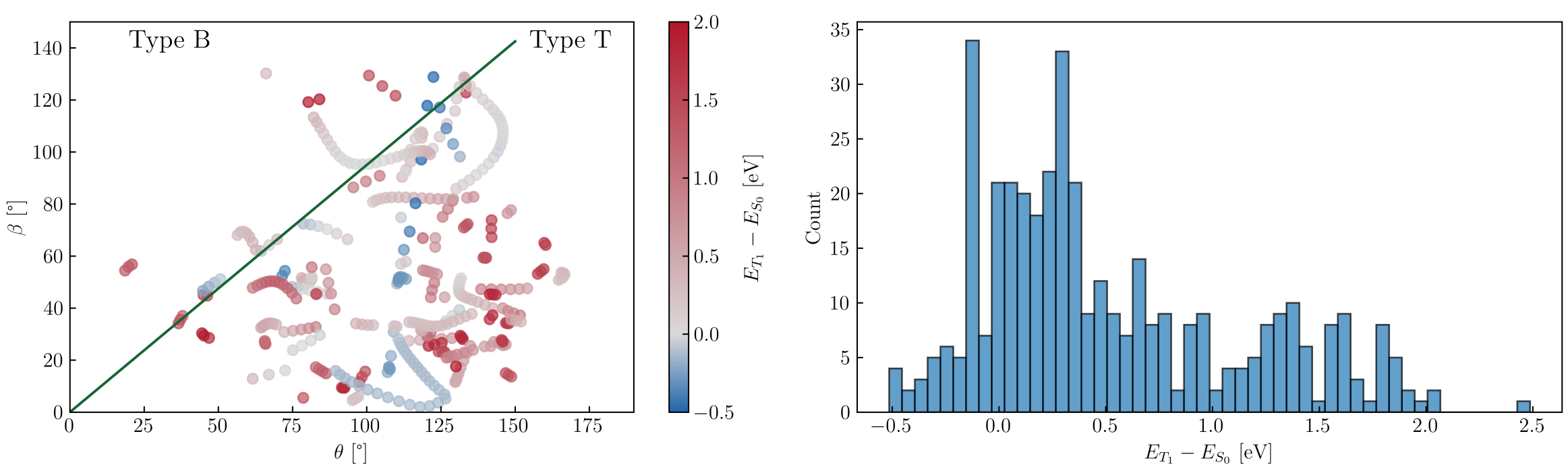

Figure S19: Projection of geometries collected from 200 MR-CISD trajectories with $\alpha = 15$ related to the increase of T_1 population and colored by $E_{T_1} - E_{S_0}$.

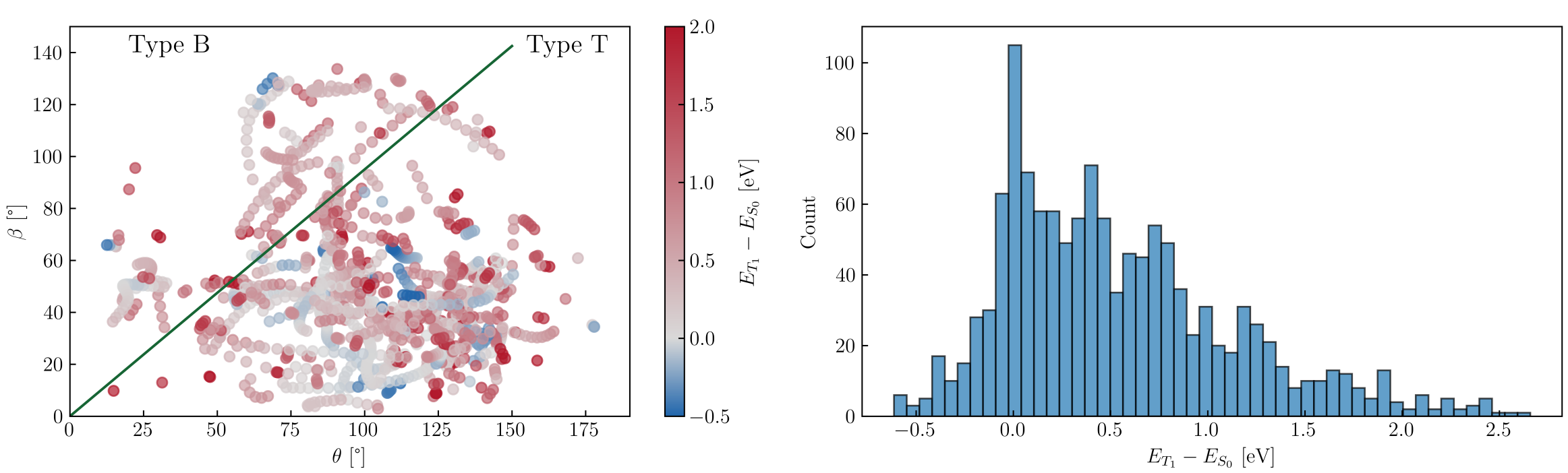

Figure S20: Projection of geometries collected from 200 MR-CISD trajectories with $\alpha = 25$ related to the increase of T_1 population and colored by $E_{T_1} - E_{S_0}$.

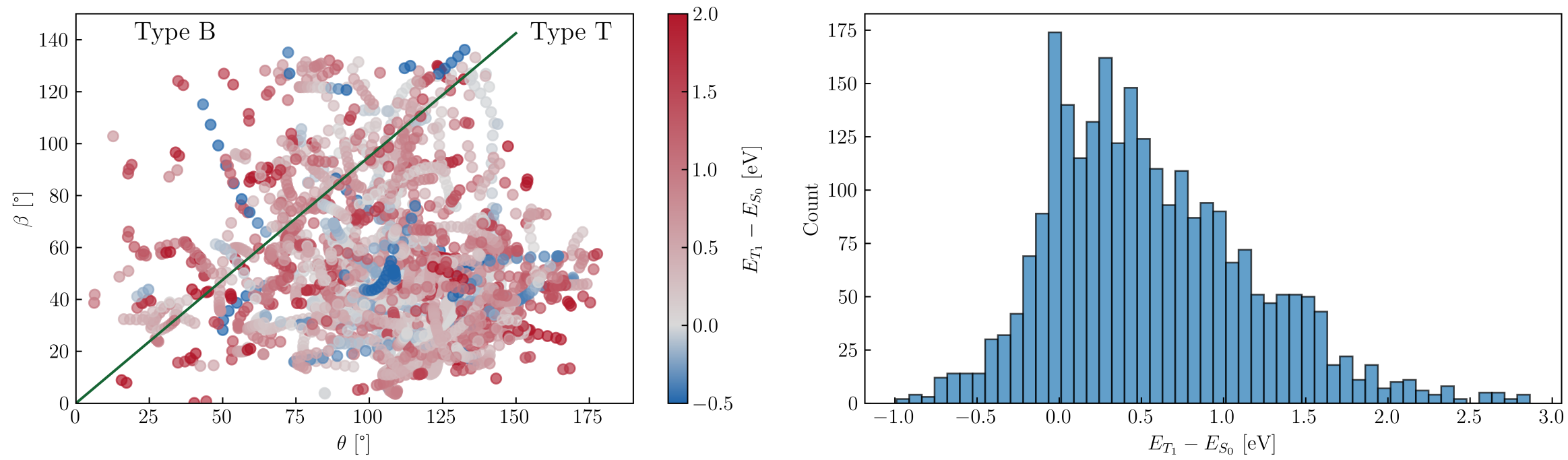

Figure S21: Projection of geometries collected from 200 MR-CISD trajectories with $\alpha = 35$ related to the increase of T_1 population and colored by $E_{T_1} - E_{S_0}$.

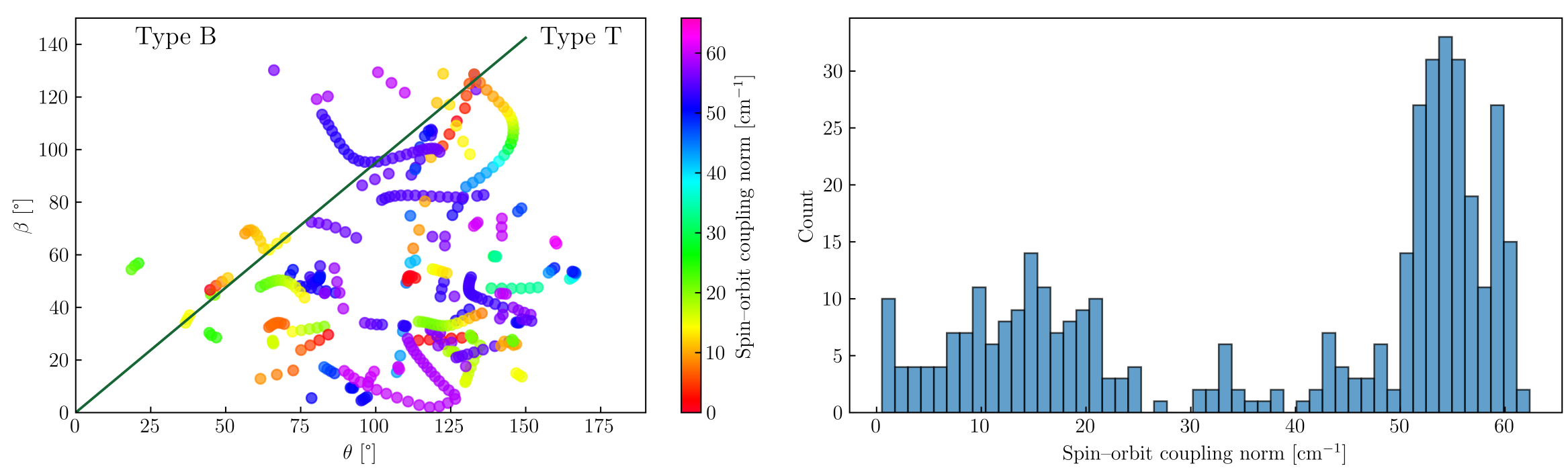

Figure S22: Projection of geometries collected from 200 MR-CISD trajectories with $\alpha = 15$ related to the increase of T_1 population and colored by SOC magnitude.

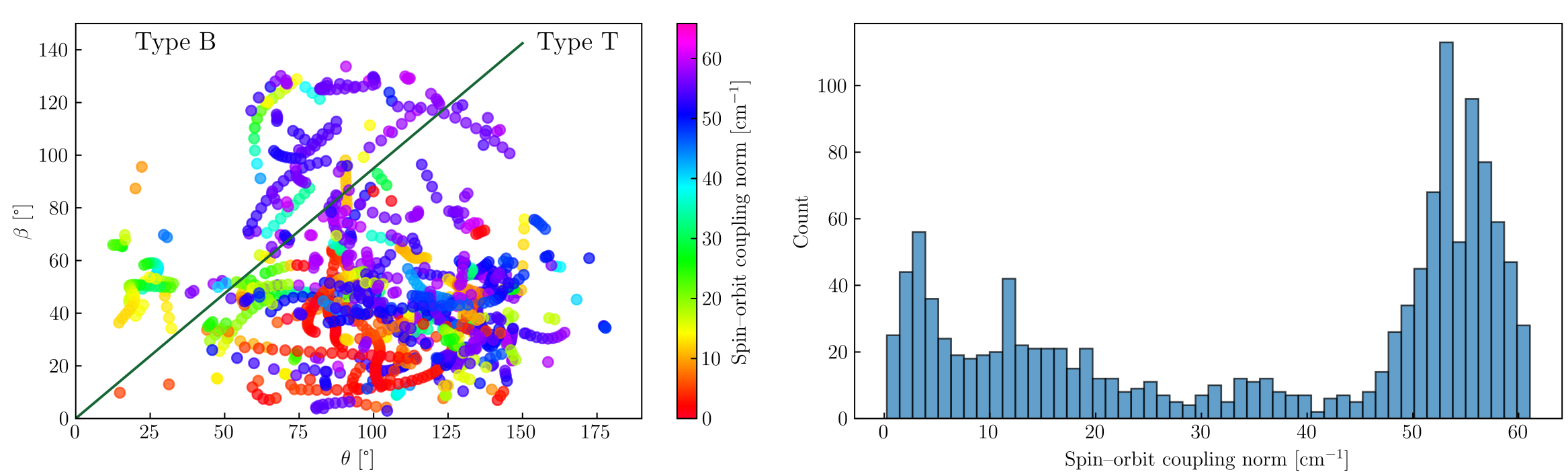

Figure S23: Projection of geometries collected from 200 MR-CISD trajectories with $\alpha = 25$ related to the increase of T_1 population and colored by SOC magnitude.

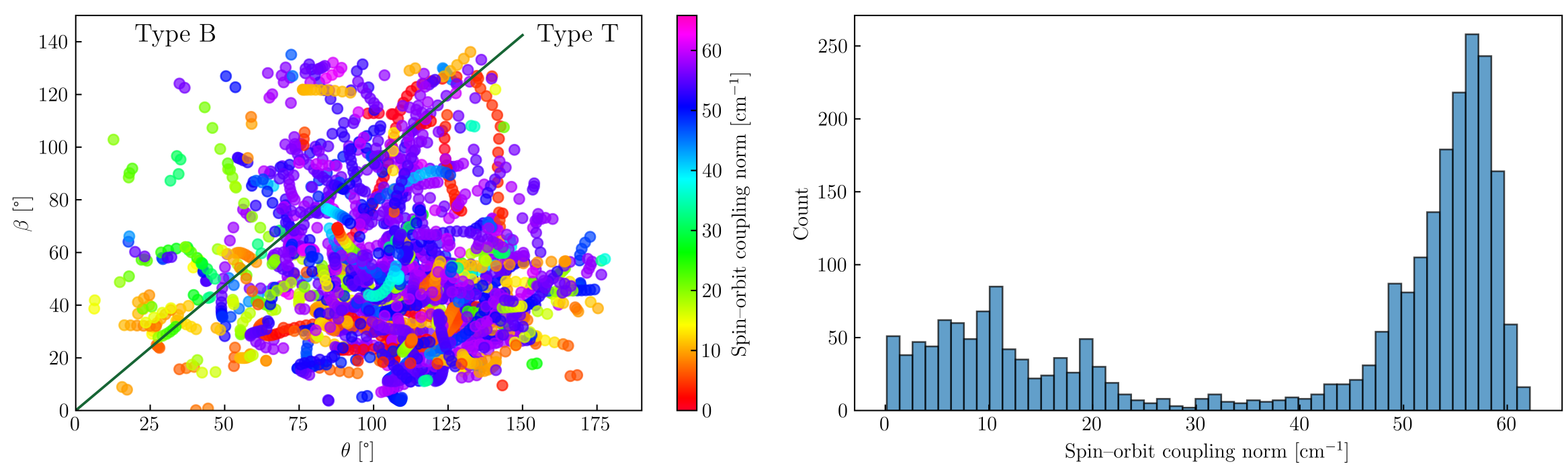

Figure S24: Projection of geometries collected from 200 MR-CISD trajectories with $\alpha = 35$ related to the increase of T_1 population and colored by SOC magnitude.

The effects of decoherence and the singlet and triplet composition of the active PES

The production dynamics was performed using ML-driven dynamics without a decoherence correction (DC). To assess its influence on the dynamics, we ran four sets of trajectories using the ML models with a scaling factor of 15 (see Fig. S25). The first set represents the trajectories reported in the main text ($|c_i|^2$ without DC). The second set uses the same initial conditions and dynamics settings, including the same random seeds ($|c_i|^2$ with DC). The third and fourth sets compare the populations from the first two sets with the singlet and triplet composition of the active PES, averaged over all trajectories. In all cases, almost indistinguishable populations are observed, providing confidence in the population plots reported in the main text.

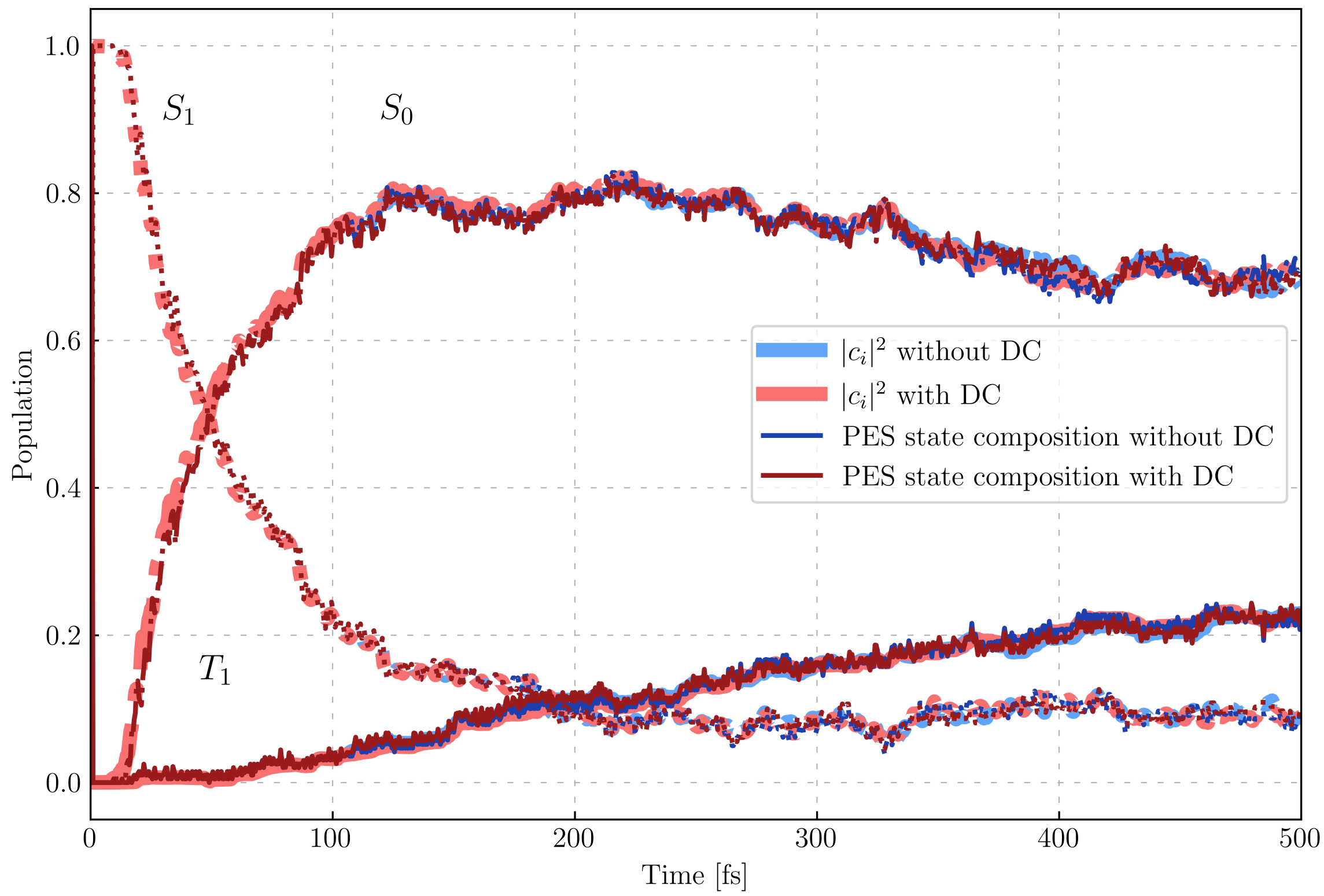

Figure S25: Effect of the decoherence correction (DC) on the population dynamics. Populations obtained with and without DC are compared with the singlet and triplet composition of the active PES. The trajectories were propagated using the ML models with a scaling factor of 15.
